# Dynamic synovial fibroblasts are modulated by NBCn1 as a potential target in rheumatoid arthritis

**DOI:** 10.1038/s12276-022-00756-6

**Published:** 2022-04-12

**Authors:** Minjeong Ji, Hee Jung Ryu, Hyeon-Man Baek, Dong Min Shin, Jeong Hee Hong

**Affiliations:** 1 Department of Physiology, College of Medicine, Gachon University, Lee Gil Ya Cancer and Diabetes Institute, 155 Getbeolro, Yeonsu-gu, Incheon, South Korea; 2 Department of Oral Biology, Yonsei University College of Dentistry, Seoul, South Korea; 3 Division of Rheumatology, Department of Internal Medicine, Gachon University Gil Medical Center, 21 Namdongdae-ro 774-gil, Nandong-gu, Incheon, South Korea; 4 Department of Health Sciences and Technology, GAIHST, Gachon University, Incheon, South Korea; 5 Lee Gil Ya Cancer and Diabetes Institute, Gachon University, 155 Getbeolro, Yeonsu-gu, Incheon, South Korea

**Keywords:** Chemotaxis, Bone, Ion channel signalling, Rheumatoid arthritis, Drug development

## Abstract

Rheumatoid arthritis (RA) is an autoimmune disease characterized by aggressive fibroblast-like synoviocytes (FLSs) and pannus formation. Various therapeutic strategies have been developed against inflammatory cytokines in RA in recent decades. Based on the migratory features of FLSs, we examined whether modulation of the migratory module attenuates RA severity. In this study, inflamed synovial fluid-stimulated FLSs exhibited enhanced migration and migratory apparatus expression, and sodium bicarbonate cotransporter n1 (NBCn1) was identified in primary cultured RA-FLSs for the first time. The NBC inhibitor S0859 attenuated the migration of FLSs induced with synovial fluid from patients with RA or with TNF-α stimulation. Inhibition of NBCs with S0859 in a collagen-induced arthritis (CIA) mouse model reduced joint swelling and destruction without blood, hepatic, or renal toxicity. Primary FLSs isolated from the CIA-induced mouse model also showed reduced migration in the presence of S0859. Our results suggest that inflammatory mediators in synovial fluid, including TNF-α, recruit NBCn1 to the plasma membrane of FLSs to provide dynamic properties and that modulation of NBCn1 could be developed into a therapeutic strategy for RA.

## Rheumatoid arthritis: A potential protein target for treatment

A protein which transports ions across cell membranes has been implicated in promoting destructive cellular activities in joints affected by rheumatoid arthritis (RA) and could become a target for new drugs to treat RA. Researchers in South Korea led by Jeong Hee Hong at Gachon University, Incheon, and Dong Min Shin at Yonsei University, Seoul, identified the protein NBCn1 in fibroblast-like synoviocytes (FLS) with response to the synovial fluid of patients’ joints affected by RA. Chemical signals associated with RA enhanced the activity of NBCn1 in FLS cells, promoting the cell migration that leads to joint damage. Mouse studies revealed that inhibiting NBCn1 activity reduced the cellular signals and interactions that damage joints, leading to less swelling and tissue destruction. Drugs that reduce NBCn1 activity could therefore offer new opportunities to treat RA.

## Introduction

Rheumatoid arthritis (RA) is a chronic inflammatory autoimmune disease that is histologically characterized by hyperplasia of fibroblast-like synoviocytes (FLSs), inflammatory cell infiltration, pannus formation, and progressive joint destruction^1,2^. The synovium is the primary site of the inflammatory process, and synovial FLSs mediate joint deterioration and destruction in RA^3–5^. The normal synovium is a thin layer^6^, while the RA synovium is in a hyperplastic state^7^. The inflamed synovium, which secretes various proinflammatory cytokines, such as tumor necrosis factor (TNF-α) and chemokines, can activate local FLSs and induce FLS hyperplasia and invasion^8^. The migration of FLSs isolated from patients with RA (RA-FLSs), which involves movement toward the inflamed joint, mediates joint destruction and cartilage degradation, which are serious symptoms of RA^9–11^.

Cellular migration is modulated by various ion channels and transporters that constitute the motile machinery^12^. It can be assumed that migratory FLSs recruit ion transporters to achieve motility. Therefore, the identification of migratory modules and mechanisms of their modulation could be used to develop a therapeutic approach for targeting RA-FLSs, which could enable more effective control of RA activity^13–17^. However, the identities of the transporters involved in FLS migration remain to be defined.

Na^+^-HCO_3_^−^ cotransporters (NBCs), encoded by the SLC4 family, are known to be intracellular pH regulators and acid-extruding transporters^18^. NBC activity affects the migration of renal epithelial cells, Madin-Darby canine kidney fibroblast cells, and lung and breast cancer cells^19–22^. Therefore, we hypothesized that modulation of the activity of NBC transporters involved in RA-FLS migration could be a therapeutic strategy to inhibit activated FLS migration. In this study, we demonstrated for the first time that enhanced FLS migration induced by the inflammatory cytokine TNF-α recruited electroneutral NBC1 (NBCn1). The movement of FLSs induced by synovial fluid from patients with RA could be attenuated by inhibiting NBC activity. Furthermore, inhibition of NBCs in a collagen-induced arthritis (CIA) mouse model was also studied to provide a new therapeutic target in RA.

## Materials and methods

### Patient enrollment and synovial fluid collection

All procedures for synovial fluid collection were performed in accordance with the Declaration of Helsinki, and this study was approved by the Institutional Review Board of Gil Medical Center (GAIRB-2019-018). The collected synovial fluid samples, whose collection was permitted by the patients, were effused from the knee joints of eight patients with RA who fulfilled the 2010 American College of Rheumatology (ACR)/European League Against Rheumatism (EULAR) classification criteria^23^. The synovial fluid (≥10 mL) was obtained by therapeutic arthrocentesis using a 30-mL syringe with a 21-gauge needle via an aseptic procedure. The obtained synovial fluid samples were centrifuged at 1500 rpm for 15 min and stored at 4 °C until use.

### HEK293T cell culture and DNA transfection

Human HEK293T cells were purchased from ATCC® (USA, CRL-3216^TM^). Cells were cultured in Dulbecco’s modified Eagle’s medium (DMEM) containing 10% fetal bovine serum (FBS) and 100 U/mL penicillin–streptomycin and incubated at 37 °C in 5% CO_2_/95% air. A human NBCn1 clone was provided by Dr. Shmuel Muallem (National Institutes of Health, Bethesda, MD, USA). DNA transfection was performed according to the manufacturer’s protocol with 200 μL of Jet prime buffer (Polyplus, USA, #B200225) mixed with 4 μL of transfection reagent (Polyplus, #21Y0910L1) for 48 h.

### Primary FLS culture

Human primary FLSs isolated from RA synovial tissues, RA-FLSs (408RA-05a) and FLSs derived from osteoarthritis (OA) patients (OA-FLSs, 408OA-05a) were purchased from Cell Application, Inc. (San Diego, CA)^24,25^. FLSs were maintained in Dulbecco’s modified Eagle’s medium (Invitrogen, 11995–065) containing 100 U/mL penicillin–streptomycin (Invitrogen, 15140–122) and 10% fetal bovine serum (FBS; Invitrogen, 16000–044) and cultured at 37 °C in 5% CO_2_/95% air. When FLSs reached 80% confluency, the cell culture medium was aspirated, and the cells were washed with Dulbecco’s phosphate-buffered saline (DPBS; Welgene, South Korea, LB001-02), followed by treatment with trypsin/ethylenediaminetetraacetic acid (EDTA) for 1 min. RA-FLSs and OA-FLSs were used within 5 passages for all experiments involving primary cultured FLSs.

### Transwell membrane migration assay

The migration assay was conducted using a Transwell-polycarbonate membrane (6.5-mm insert, 8.0-μm pore size). The inserts were filled with 200 μL of FLSs (5 × 10^4^ cells; Cell Applications, Inc., San Diego, CA, USA) containing 1% FBS and reagents. The bottom chamber was treated with 10 ng/mL TNF-α dissolved in 500 μL of DMEM at different pH values, and synovial fluid samples from RA patients were diluted with DMEM at a 1:1 ratio and then incubated for 6 h. Then, the DMEM in the bottom chamber was removed, chilled methanol was added, and the insert membranes were soaked for 1 min at −20 °C. The chilled methanol was removed, and the plate was washed three times with phosphate-buffered saline (PBS). A DAPI solution was mixed with distilled water and loaded into the bottom chamber. The plate was incubated for 30 min in the dark. Then, the medium was carefully removed from the insert. Distilled water (DW) was added to the bottom chamber at room temperature, and DAPI fluorescence was measured at 405 nm using an LSM 700 Zeiss confocal microscope (Fluoview, Carl Zeiss, Germany). FLS migration was determined from the number of nuclei stained with DAPI on the Transwell membrane.

### Immunostaining of the insert membrane in a Transwell culture system

After the migration assay, the insert was restored for immunostaining. The membrane of the insert was cut, isolated, and immunostained using an anti-NBCn1 antibody (Abcam, Cambridge, UK, ab82335). The isolated membrane was added to a 50 mM glycine solution for 10 min at 4 °C, and then a 5% blocking solution was added and incubated for 1 h at room temperature in the dark. The antibody was diluted at 1:100 and incubated overnight at 4 °C. The secondary antibody, rhodamine-tagged goat anti-rabbit immunoglobulin G (IgG), was added and incubated for 1 h at room temperature. Finally, the membrane was carefully attached and mounted on a glass slide with DAPI-included Fluoromount-G^TM^ (Electron Microscopy Sciences, Hatfield, PA), and fluorescence images were acquired using an LSM 700 Zeiss confocal microscope (Fluoview) and analyzed with ZEN software.

### Measurement of Na^+^-HCO_3_^−^ cotransporter (NBC) activity

For analysis of pH_i_ measurement-based NBC activity, changes in the pH_i_ of FLSs were measured with 2′-7′-bis-(carboxyethyl)-5-(and-6)-carboxyfluorescein (BCECF-AM; Teflabs, Austin, TX, USA; #0061) at the dual excitation wavelengths of 440 and 495 nm and an emission wavelength of 530 nm. FLSs attached to coverslips were loaded into the chamber with 6 µM BCECF-AM in the presence of 0.05% Pluronic F-127 for 15 min at room temperature. The dye-loaded FLSs were perfused with a physiological salt solution (the composition was previously described^26^) for at least 5 min to stabilize the fluorescence before measuring pH_i_ at 37 °C. NBC activity was measured by incubating the cells with CO_2_-saturated HCO_3_^−^-buffered medium containing 5-(N-ethyl-N-isopropyl) amiloride (EIPA) (Sigma, 1154-25-2), followed by acidification with Na^+^-free HCO_3_^−^-buffered medium. The emitted fluorescence was monitored using a CCD camera (Photometrics) attached to an inverted microscope (Olympus, Japan) and analyzed with a MetaFluor system (Molecular Devices). All BCECF fluorescence images were obtained at 1-s intervals, and the background fluorescence signal was subtracted from the raw background signals at each wavelength. NBC activity was determined from the derivatives of the initial enhanced slopes from the first 1 min of pH_i_ increase in the Na^+^-free HCO_3_^−^-buffered medium, as previously reported^27^.

### MTT assay

FLSs (~5,000 cells) were cultured in 96-well plates for 24 h after adding the indicated reagent and medium (S0859, DMEM under different pH conditions) for 6 h. Tetrazolium bromide (MTT; 2 mg; Merck, Burlington, MA, USA; 298-93-1) dye was mixed with 1 mL of PBS, and the cells were treated with 100 μL of MTT dye and incubated for 2 h in the dark. The medium was carefully aspirated from the plate. Then, 100 µL of DMSO was added to the plates, and the absorbance was measured at 570 nm using a fluorescence microplate reader (VICTOR X3, PerkinElmer, Waltham, MA, USA).

### Total RNA extraction and quantitative real-time polymerase chain reaction (RT–qPCR)

Total RNA was isolated from primary FLSs using a Hybrid-Ribo^Ex^ extraction system (Gentaur, Belgium) following the manufacturer’s instructions. RNA was quantitated using an ND-1000 spectrophotometer (Thermo Fisher, Waltham, MA) and then amplified using a cDNA synthesis kit from Enzynomics (Daejeon, South Korea) according to the manufacturer’s instructions. RT–qPCR was performed with Power Up^TM^ SYBRTM Green Master Mix (Applied Biosystems^®^, Waltham, MA, USA; A25741) and primers using a QuantStudioTM3 RT–PCR system (Applied Biosystems^®^, A28567). The primers used are listed in Supplementary Table 1. The RT–qPCR cycling protocol was as follows: UDG activation at 50 °C for 2 min, Dual-Lock DNA polymerase at 95 °C for 2 min, denaturation at 95 °C for 15 s, annealing at 55 °C for 15 s, and extension at 72 °C for 1 min.

### pH calibration

The intracellular pH (pH_i_) value, which was measured with BCECF-AM, was calculated using calibration curves as described previously^28,29^. Briefly, a calibration solution with specific pH-dependent stimulatory conditions (5.5–8.5, 0.5 interval) was applied to FLSs attached to coverslips for pH calibration. The FLSs were incubated with each calibration solution for 5 min at room temperature. The following equation was used to generate the pH calibration curve: pH = 6.97–log([7.19-R_max_]/[R_min_−2.41]) (pKa of BCECF, 6.97; R, ratio value of BCECF; R_max_, maximum ratio; R_min_, minimum ratio). The changes in intracellular pH (ΔpH_i_) values were calculated using the BCECF fluorescence ratio and are represented by the calibration curve (Supplementary Fig. 1).

### Collagen-induced arthritis (CIA) mouse model

All experimental animal procedures were performed in accordance with the Gachon University guidelines and were approved by the Animal Care and Use Committee of Gachon University (LCDI-2019-0139). DBA/1 (6 weeks, male, 22–25 g) mice were purchased from Orient Bio (Seongnam, South Korea). CIA was induced with 4 μg of bovine type II collagen/g (weight) (Chondrex, Inc., Woodinville, WA, USA; 20021) dissolved in complete Freund’s adjuvant (Sigma Aldrich, Saint Louis, MO, USA; F5881) at a 1:1 ratio administered via intradermal injection for the first immunization. After 2 weeks, 4 μg of bovine type II collagen/g (weight) was dissolved in incomplete Freund’s adjuvant (Sigma, F5506) at a 1:1 ratio and administered as a booster injection.

### Treatment of CIA

CIA was induced through two collagen injections. Animals were divided into CIA, CIA + S0859, S0859, CIA + vehicle (DMSO), and vehicle (DMSO) groups. Six weeks after the first collagen injection, S0859 (Cayman, Ann Arbor, MI, USA; No. 18497) dissolved in DMSO (Bio Life Solutions, Bothell, WA, USA; 19128) was diluted in saline and injected via the mouse tail vein at a concentration of 800 ng of S0859/g (weight). Two weeks after the first S0859 injection, a second dose of S0859 at the same concentration was injected, and the physical and behavioral abnormalities of the mice were monitored during treatment. Thirty mice were enrolled in the control or vehicle group, and 45 mice were enrolled for CIA modeling. The mice in all groups were sacrificed 14 days after the second S0859 injection. The serum and hind paws of the animals in all groups were collected for analysis.

### Three-dimensional micro-computed tomography (CT)

Ten weeks after the development of RA, all control and CIA-induced mice were sacrificed. All hind paws were dissected, divided into the control, CIA, and S0859-injected groups, and treated with 10% neutral buffered formalin (NBF) (Biosesang, South Korea) for 1 h. Micro-CT was conducted on the ankle joint of the hind paw using a Quantum FX Micro-CT (Perkin Elmer, Waltham, MA) with the following conditions: exposure for 2 min at 90 kV, 160 μA, and field-of-view of 24 mm.

### Determination of the paw score and thickness

Mouse paws were scored for arthritis using a scoring system ranging from 0 to 4 for each paw^30,31^ in a blinded manner: normal paw, 0; one toe inflamed and swollen, 1; more than one toe inflamed and swollen, 2; entire paw inflamed and swollen, 3; and severe inflammation and swelling of the ankylosed paw, 4 (on the scale of 0 to 16). Two weeks after the second S0859 injection, the mice were anesthetized, the paw score was measured by two independent blinded observers, and the thickness of the hind paw was measured using a caliper.

### Magnetic resonance image (MRI) acquisition

MRI data acquisition was conducted on a 9.4-tesla (T) Bruker BioSpec horizontal bore dedicated animal scanner (Bruker Biospin, Ettlingen, Germany) equipped with a gradient system (660 mT/m). For radio frequency (RF) excitation, a quadrature volume resonator (inner diameter, 114 mm; Bruker Biospin) was used. For signal reception, a four-channel mouse surface coil (Bruker Biospin) was applied. All image data acquisition was performed at the Cell to In Vivo Imaging Core Facility Research Center (CII, Lee Gil Ya Cancer and Diabetes Institute, Gachon University, Incheon, South Korea). MRI data were acquired using Paravision 6.0 software. The pulse sequence used for this acquisition was T1-weighted MRI (3D fast low angle shot magnetic resonance imaging (FLASH, spin echo sequence with a repetition time = 50 msec, echo time = 8 msec, flip angle = 20°, bandwidth = 27.8 kHz, slice thickness = 15.6 mm, field of view = 1.2 × 1.2 cm, matrix = 120 × 120 × 156, resolution = 100 × 100 × 100 µm, 2 averages and resulting in a total acquisition time of 34 min 36 s)) and T2-weighted MRI (3D spin echo technique (TurboRARE), repetition time = 2000 msec, echo time = 33 msec, flip angle = 90°, bandwidth = 119.1 kHz, slice thickness = 15.6 mm, field of view = 1.2 × 1.2 cm, matrix = 120 × 120 × 156, resolution = 100 × 100 × 100 µm, 1 average and resulting in a total acquisition time of 40 min 8 s).

### Immunohistochemistry (IHC)

The mouse hind paws, including the ankle joints, were harvested, and the skin was removed. After fixation with 10% NBF for 24 h, the joints were decalcified in RapidCal Immuno^TM^ (BBC Biochemical) for 2 weeks. Subsequently, the joints were fixed with 10% NBF for 24 h. The ankle joints were embedded in paraffin, and coated slides were stained with hematoxylin and eosin (H&E). IHC was performed using an anti-NBCn1 antibody at a 1:1000 dilution to evaluate the control, CIA, and S0859-injected groups. All IHC analyses were performed by using a Panoramic SCAN II (3DHISTECH, Hungary) at the Core Facility for Cell to In Vivo (Bio) Imaging (Lee Gil Ya Cancer and Diabetes Institute, South Korea). The number of immune cells that infiltrated the inflamed area^32^ was measured using a Leica DM2500 microscope (Leica Microsystems, Germany).

### Enzyme-linked immunosorbent assay (ELISA)

Under anesthesia, all blood samples were collected directly from the heart using a 1-cc syringe (Kovax-syringe, South Korea). Blood samples used in a complete blood count (CBC) test were stored in a vacutainer containing EDTA immediately after collection. The hemoglobin (HGB, g/dL), mean corpuscular hemoglobin (MCH, pg), hematocrit (HCT, %), mean corpuscular volume (MCV, fL), mean corpuscular hemoglobin concentration (MCHC, g/dL), platelet (PLT, cells/μL), red blood cell (RBC, cells/mL), white blood cell (WBC, cells/mL), eosinophil (EOS, %), neutrophil (NEU, %), and lymphocyte (LYM, %) levels were assessed using the CBC test. The remainder of each sample was centrifuged at 1500 rpm for 15 min, and the supernatants were aspirated and transferred to fresh EP tubes. An S0859 toxicity test was conducted using hepatic and renal panels. The levels of alanine aminotransferase (ALT, U/L), aspartate aminotransferase (AST, U/L), and total bilirubin (T-Bili, U/L) were measured to evaluate liver function. Blood urea nitrogen (BUN, mg/dL) was used to evaluate renal function. The levels of TNF-α (R&D Systems, Minneapolis, MN, No. MTA00B), IL-6 (R&D Systems, No. M6000B), and IL-1β (R&D Systems, No. MLB00C) were measured using a mouse Quantikine^®^ ELISA kit (R&D Systems) following the manufacturer’s instructions.

### Isolation of FLSs from CIA mice

The skin around the knee was removed from sacrificed CIA mice. The synovium was harvested, transferred to a 60-mm petri dish, washed with PBS, and minced using surgical scissors. The minced tissues were incubated with 1% type IV collagenase at 37 °C for 1 h. The tissue samples were vortexed and centrifuged at 300 g for 5 min. The supernatants were discarded, and DMEM containing 10% FBS and 1% penicillin–streptomycin was added to release the cells. Then, the cells were cultured at 37 °C and 5% CO_2_ to allow the cells to reach adequate confluence.

### Fluorescence-activated cell sorting (FACS)

For FACS analysis, all cells were dissociated into single cells in PBS supplemented with 1% FBS. To identify FLSs, primary cultured FLSs from CIA mice were incubated with an FLS marker-specific antibody, anti-CD90.2 (Invitrogen, 13-0903-85)^33^, for 30 min at 4 °C. The incubated cells were washed with PBS three times, centrifuged at 1,500 rpm for 5 min, and then incubated with a FITC-conjugated anti-rabbit IgG antibody for 30 min at 4 °C. After three washes with PBS, the centrifuged cells were suspended in PBS supplemented with 1% FBS and then passed through a 35-μm filter tube. The cell population was analyzed using a flow cytometer (BD LSRII, BD Biosciences), and the data were analyzed using FlowJo software (FlowJo, OR, USA). The population of CD90.2-positive cells was determined using FITC fluorescent dots against the negative control.

### Statistical analysis

Results are expressed as the mean ± standard error of the mean (SEM). Statistical differences between the mean values of two sample groups were analyzed using Student’s *t* test. The statistical significance in each experiment was determined by analysis of variance (*P < 0.05, **P < 0.01, and ***P < 0.001).

## Results

### RA synovial fluid samples stimulated RA-FLS migration and enhanced NBC activity

**Fig. 1:**
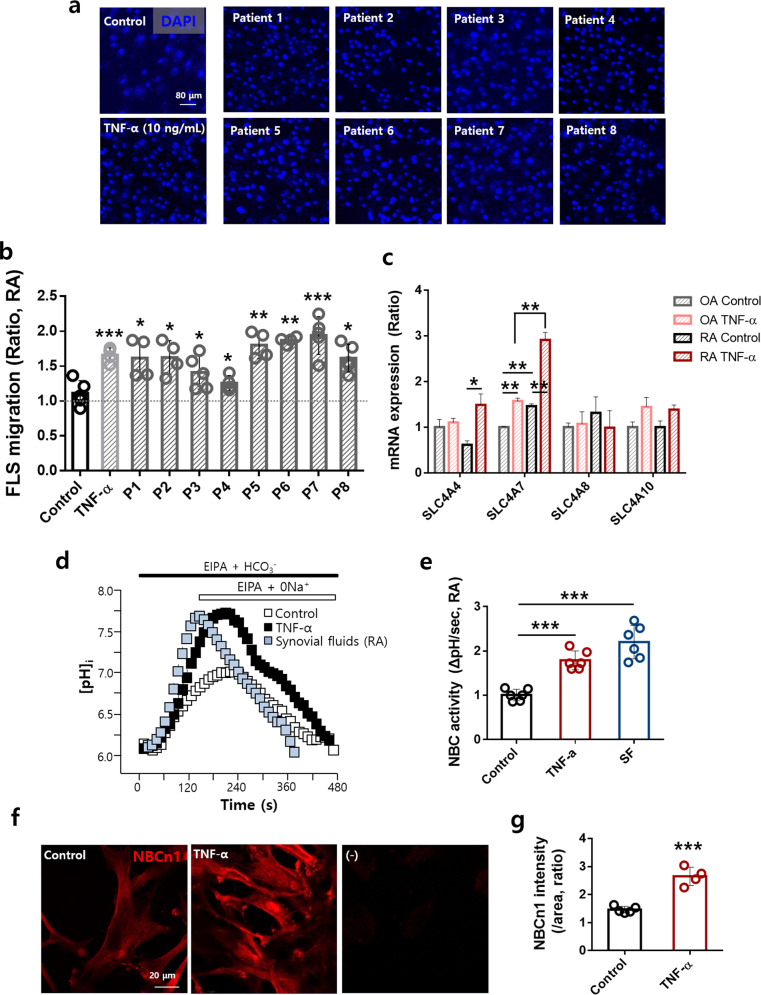
RA synovial fluid samples stimulated RA-FLS migration and enhanced NBC activity. **a** FLS migration assay performed with 10 ng/mL TNF-α or RA-synovial fluid. Immunofluorescence staining with DAPI (blue). The scale bar represents 80 μm. **b** Analysis of the total DAPI intensity to assess FLS migration. P: Patient. Bars represent the mean ± SEM (*n* = 4, *p < 0.05, **p < 0.01, and ***p < 0.001). * shows a statistically significant difference between each sample and the control. **c** The relative mRNA levels of *SLC4A4, SLC4A7, SLC4A8*, and *SLC4A10* in RA-FLSs or OA-FLSs stimulated with TNF-α. **d** NBC activity was assessed by measuring changes in the pH_i_ values of Transwell-cultured migrated FLSs following treatment with 10 ng/mL TNF-α or RA-synovial fluids for 6 h. **e** The bars represent the mean ± SEM (*n* = 6, ***p < 0.001). **f** Immunofluorescence staining for NBCn1 (red) in migrated FLSs treated with 10 ng/mL TNF-α for 6 h. The scale bar represents 20 μm. (−), negative control. **g** Analysis of the normalized intensity (total intensity/measuring area) of NBCn1. Bars represent the mean ± SEM (*n* = 4–5, ***p < 0.001).

The clinical characteristics of the patients with RA included in this study are shown in Table 1. We hypothesized that the dynamic movement of FLSs was caused by inflammatory signals in the synovial fluid. To evaluate the migratory ability of FLSs stimulated with synovial fluid from RA patients, we used the Boyden Transwell system. FLSs were seeded in the upper chamber, and diluted RA-synovial fluid was placed in the lower chamber. The synovial fluid samples were diluted with cell culture medium to reduce viscosity and achieve contact with the membrane of the upper chamber. We focused on the modulation of RA-FLS migration because FLSs were exposed to various proinflammatory cytokines from inflammatory cells, such as monocytes or macrophages. Among the cytokines, TNF-α was used as a positive control signaling molecule for stimulation. TNF-α-stimulated RA-FLSs migrated 1.5-fold more than control cells, and the synovial fluid samples from patients also showed a 1.64-fold increase in RA-FLS migration (Fig. 1a, b). NBCs are considered an effective module for cellular migration^19,20,34^. NBCs are encoded by several members of the solute carrier family *SLC4A*^35^, and NBC activity is derived from various splice variants of NBCs. Based on the mRNA expression patterns of RA-FLSs and OA-FLSs, the expression of *SLC4A7*, the gene encoding NBCn1, was found to be dramatically enhanced by TNF-α stimulation in RA-FLSs (Fig. 1c). To identify the migratory module of FLSs involving NBCs, NBC activity was measured in FLSs using the BCECF-based pH-indicating technique in the presence of a dominant pH regulator, the Na^+^-H^+^ exchanger (NHE) blocker EIPA^36^, and stimulation with TNF-α or RA synovial fluid. The NBC activity of FLSs was enhanced by stimulation with RA synovial fluid, similar to the enhancement achieved with TNF-α (Fig. 1d, e). To evaluate the involvement of NBCn1, RA-FLSs in the TNF-α- or RA synovial fluid-stimulated bottom chamber were immunostained for NBCn1. However, the immunostaining of cell-attached Transwell membranes was limited under the synovial fluid-stimulated conditions because the synovial fluid turned the medium in the bottom chamber into a gelatinous material. Synovial fluid was replaced with TNF-α due to experimental limitations such as its high viscosity. Therefore, the bottom chamber was stimulated with TNF-α. The intensity of NBCn1 immunostaining in RA-FLSs was enhanced by TNF-α stimulation (Fig. 1f, g). The expression of NBCn1 in the plasma membrane of RA-FLSs was enhanced. The changes in pH were calibrated, followed by plotting of the pH calibration curve (Supplementary Fig. 1). These results suggest that the synovial fluid of patients with RA stimulates RA-FLS migration and enhances NBCn1 expression and activity.

**Table 1:** Characteristics of patients with RA.

Patient	Sex	Age (yrs)	Disease duration (yrs)	RF titer (U/mL)	Anti-CCP titer (U/mL)	WBC count of the knee joint synovial fluid (/μL)	ESR (mm/h)	CRP (mg/dL)	Tender joint count	Swollen joint count	VAS general health patient (mm)	DAS28-CRP	DAS28-ESR
1	M	67	7	124	649	26420	55	15.1	14	18	58	6.9	6.9
2	F	72	13	10	1790	6320	32	0.38	2	2	31	3.2	4.1
3	F	58	2.5	8.3	2.1	—	7	0.11	2	2	13	2.6	2.7
4	F	59	4.5	68	200	5820	56	2.38	1	1	28	3.4	4.1
5	F	59	15	—	—	127878	24	10.32	1	1	30	3.9	3.5
6	F	65	3	43	>200	10870	26	5.18	4	3	36	4.5	4.4
7	F	82	8	27	238	22100	66	3.98	1	1	28	3.5	4.2
8	F	50	3	61	>200	14850	13	0.24	1	1	23	2.5	3.0

*RF titer* Rheumatoid factor titer, *Anti-CCP titer* Anti-cyclic citrullinated peptide titer, *WBC* White blood cell, *ESR* Erythrocyte sedimentation rate, *CRP* C-reactive protein, *VAS* Visual analog scale, *DAS* Disease activity score.

### The NBC inhibitor S0859 attenuated RA-FLS migration

**Fig. 2:**
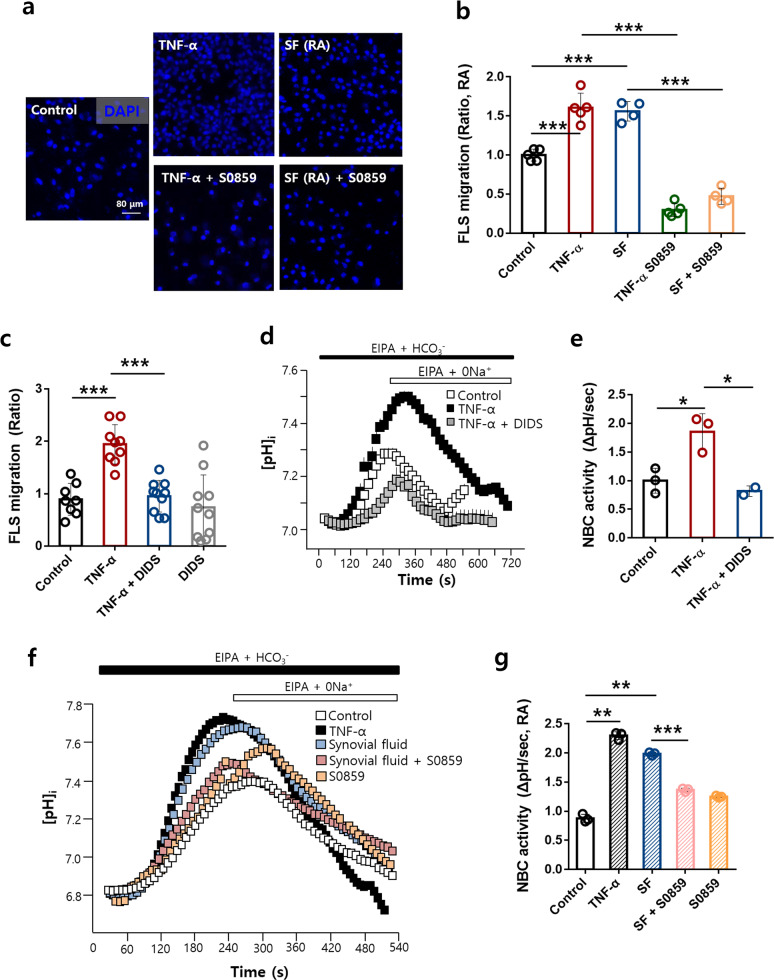
The NBC inhibitor S0859 attenuated the migration of RA-FLSs. **a** Immunofluorescence staining with DAPI (blue) after adding 10 ng/mL TNF-α or synovial fluid (SF) to the bottom chamber and 20 μΜ S0859 to the upper chamber for 6 h. The scale bar represents 80 μm. **b** Analysis of the total DAPI intensity to assess RA-FLS migration. Bars represent the mean ± SEM (*n* = 4–5, ***p < 0.001). **c** Migration assay performed with RA-FLSs stimulated with 10 ng/mL TNF-α with or without 500 μΜ DIDS. The bars represent the mean ± SEM (*n* = 8–10, ***p < 0.001) **d** NBC activity was assessed by measuring changes in the pH_i_ values of Transwell-cultured migrated RA-FLSs treated with 10 ng/mL TNF-α with or without DIDS for 6 h. **e** The bars represent the mean ± SEM (*n* = 2–3, *p < 0.05). **f** NBC activity was assessed by measuring changes in the pH_i_ values of Transwell-cultured migrated RA-FLSs treated with 10 ng/mL TNF-α or synovial fluid from patients with RA with or without S0859 for 6 h. **g** The bars represent the mean ± SEM (*n* = 3, **p < 0.01 and ***p < 0.001). SF synovial fluid.

To evaluate the role of NBCs in RA-FLSs, the NBC inhibitor S0859 was applied^34,37^. We used 20 μM S0859, independent of cell viability (Supplementary Fig. 2). The TNF-α- and synovial fluid-stimulated migration of RA-FLSs was significantly reduced by treatment with S0859 (Fig. 2a, b). We also confirmed the involvement of NBCs through use of the nonspecific inhibitor of bicarbonate transporters 4,4′-diisothiocyano-2,2′-stilbenedisulfonic acid (DIDS)^38^. The TNF-α-stimulated cellular migration and NBC activity of RA-FLSs were inhibited by DIDS treatment (Fig. 2c–e, Supplementary Fig. 3). Next, we investigated whether the NBC activity of RA-FLSs was modulated by S0859 under synovial fluid-stimulated conditions. The synovial fluid-stimulated NBC activity of RA-FLSs was inhibited by treatment with S0859 (Fig. 2f, g). The inhibitory effect of S0859 on NBC activity was also confirmed in NBCn1-overexpressing HEK293T cells (Supplementary Fig. 4). These results indicated that the NBC inhibitor S0859 inhibited cellular migration and NBC activity in RA-FLSs.

### The acidic pH-stimulated migration of RA-FLSs was attenuated by S0859

**Fig. 3:**
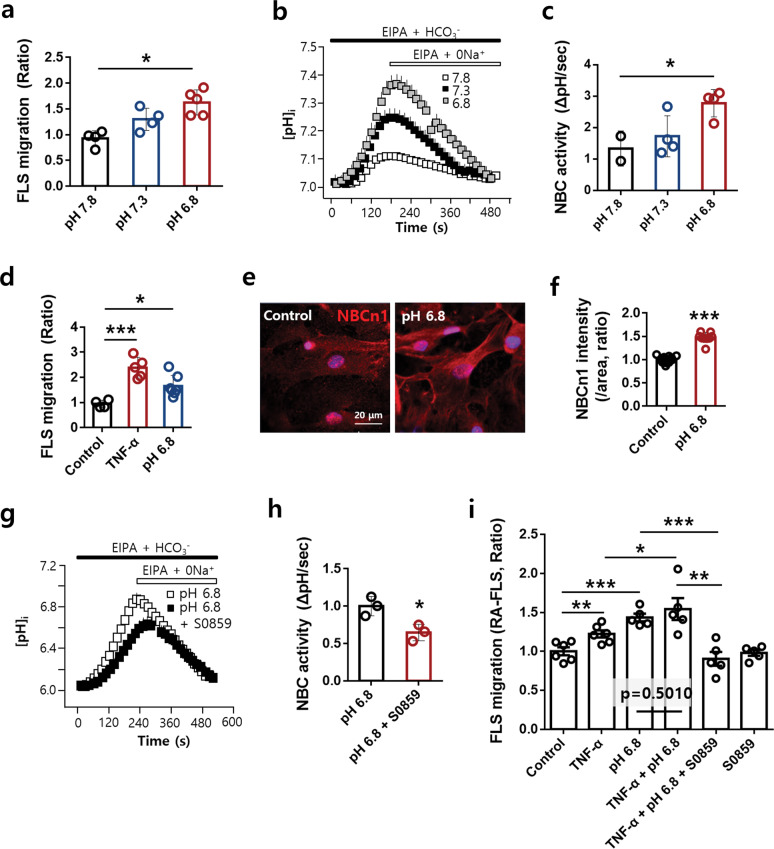
The acidic pH-stimulated migration of RA-FLSs was attenuated by S0859. **a** RA-FLS migration was measured in pH 7.8-, 7.3-, and 6.8-conditioned media in the Transwell culture system. Analysis of the total DAPI intensity to assess FLS migration. The bars represent the mean ± SEM (*n* = 4–5, *p < 0.05). **b** NBC activity was assessed by measuring changes in the pH_i_ values of Transwell-cultured migrated RA-FLSs cultured in pH 7.8-, 7.3-, or 6.8-conditioned medium for 6 h. **c** The bars represent the mean ± SEM (*n* = 2–4, *p < 0.05). **d** RA-FLS migration in TNF-α- and pH 6.8-conditioned media was measured with the Transwell culture system. Analysis of the total DAPI intensity to assess FLS migration. The bars represent the mean ± SEM (*n* = 4–5, *p < 0.05, and ***p < 0.001). **e** Immunofluorescence staining for NBCn1 (red) and DAPI staining (blue) in migrated FLSs treated with pH 6.8-conditioned medium for 6 h. The scale bar represents 20 μm. **f** Analysis of the normalized intensity (total intensity/measuring area) of NBCn1. The bars represent the mean ± SEM (*n* = 10, ***p < 0.01). **g** NBC activity was assessed by measuring changes in the pH_i_ values of Transwell-cultured migrated RA-FLSs cultured in pH 6.8-conditioned medium with S0859 for 6 h. **h** The bars represent the mean ± SEM (*n* = 3, *p < 0.05). **i** FLS migration was assessed by treating the bottom chamber with 10 ng/mL TNF-α and pH 6.8-conditioned medium and the upper chamber with 20 μΜ S0859, an NBC inhibitor. Analysis of the total DAPI intensity to assess FLS migration. The bars represent the mean ± SEM (*n* = 5, *p < 0.05, **p < 0.01, and ***p < 0.001).

A high level of hydrogen ions has been reported in the inflamed synovial fluid of patients with RA^39^, and an acidic extracellular pH favors cell migration and invasion^40^. Thus, we evaluated the effect of extracellular pH on FLS migration and the involvement of NBCn1. The migration and NBC activity of RA-FLSs were increased at an acidic pH (Fig. 3a–c). An acidic condition (pH 6.8) in the bottom chamber enhanced RA-FLS migration in response to TNF-α stimulation (Fig. 3d, Supplementary Fig. 5). Acidic extracellular conditions did not affect cell viability (Supplementary Fig. 6), and TNF-α stimulation did not affect cellular pH levels in a dose- or time-dependent manner (Supplementary Fig. 7). NBCn1 expression was also increased at pH 6.8 (Fig. 3e, f). The NBC activity of RA-FLSs, which was enhanced at pH 6.8, was inhibited by S0859 treatment (Fig. 3g, h, Supplementary Fig. 8). Additionally, inflamed synovial fluid has an acidic pH and contains TNF-α. Stimulation with an acidic condition (pH 6.8) or TNF-α enhanced the migration of RA-FLSs; on the other hand, co-stimulation with both the pH 6.8 condition and TNF-α revealed a mild or non-additive effect on the migration of RA-FLSs (Fig. 3i, Supplementary Fig. 9). S0859 treatment also produced an inhibitory effect in the co-stimulated condition (Fig. 3i, Supplementary Fig. 9). These results indicated that the effects of an acidic condition and TNF-α stimulation on the migration of RA-FLSs can be attenuated by S0859.

### S0859 attenuated the mRNA expression of pathological factors of RA in RA-FLSs

**Fig. 4:**
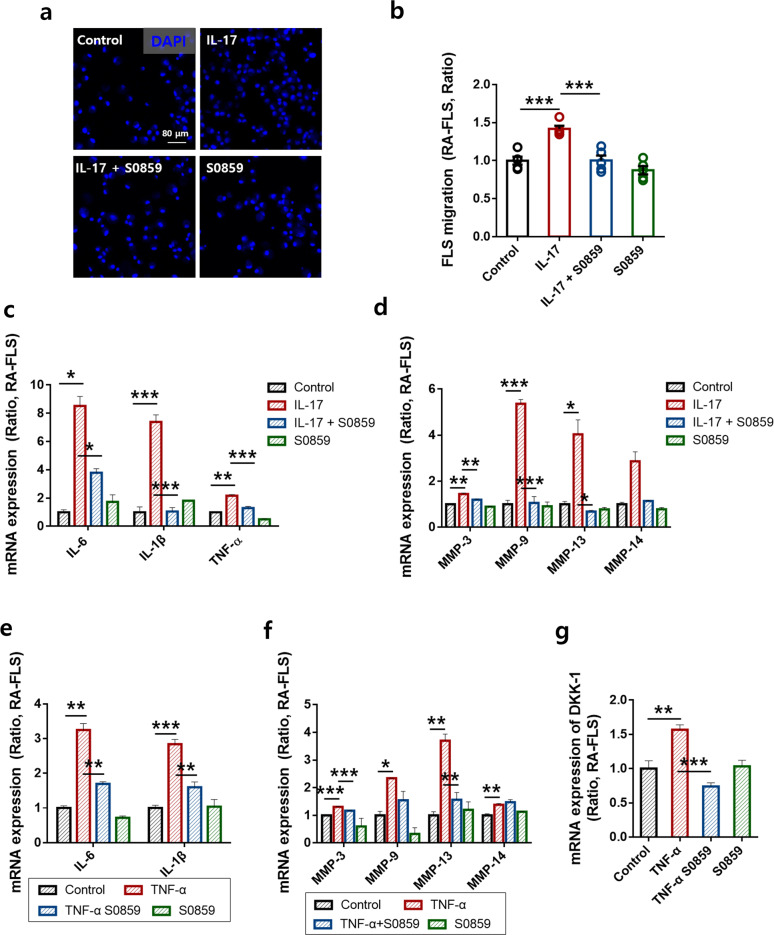
S0859 attenuated the mRNA expression of pathological factors of RA in RA-FLSs. **a** Immunofluorescence staining with DAPI (blue) after adding 10 ng/mL IL-17 to the bottom chamber and 20 μΜ S0859 to the upper chamber for 6 h. The scale bar represents 80 μm. **b** Analysis of the total DAPI intensity to assess RA-FLS migration. The bars represent the mean ± SEM (*n* = 5, ***p < 0.001). The mRNA levels of inflammatory IL cytokines and MMPs in RA-FLSs after IL-17 stimulation **c**, **d** or TNF-α stimulation **e**, **f** with or without S0859. MMPs (MMP-3, MMP-9, MMP-13, and MMP-14). **g** The mRNA levels of DKK-1 in RA-FLSs after TNF-α stimulation with and without S0859-. The bars represent the mean ± SEM (*n* = 5, *p < 0.05, **p < 0.01, and ***p < 0.001).

To verify the inhibitory role of S0859 in the pathological stimulation mediated by RA-synovial fluid, we chose IL-17, which is found in the synovial fluid of patients with RA^41,42^. The IL-17-stimulated migration of RA-FLSs was reduced by treatment with S0859 (Fig. 4a, b). The mRNA expression of inflammatory cytokines, such as TNF-α, IL-6, and IL-1β, and migratory mediators, such as MMP-3, MMP-9, and MMP-13 was reduced in S0859-treated FLSs stimulated with IL-17 (Fig. 4c, d). The mRNA expression of IL-6, IL-1β, MMP-3, and MMP-13 was reduced in S0859-treated FLSs stimulated with TNF-α (Fig. 4e, f). The inhibitory effect of S0859 on TNF-α-stimulated MMP expression was not statistically significant for MMP-9 or MMP-14 (Fig. 4f). To evaluate the therapeutic potential of S0859 in joints, the mRNA level of dickkopf-1 (DKK-1) was examined. DKK-1 is a regulatory factor involved in joint remodeling and a diagnostic factor in the pathogenesis of RA^43,44^. S0859 treatment attenuated the mRNA level of DKK-1 in TNF-α-stimulated RA-FLSs (Fig. 4g).

### S0859 attenuated structural abnormalities in the paws and bones of CIA mice

**Fig. 5:**
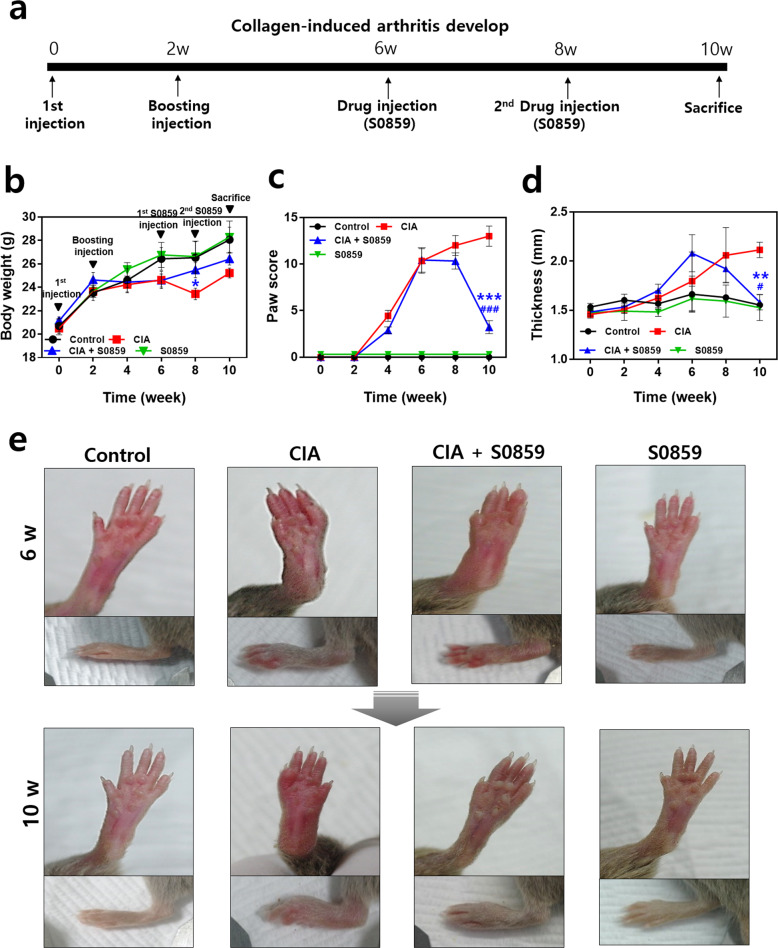
S0859 attenuated structural abnormalities in the paws and bones of CIA mice. **a** Schematic diagram of arthritis development in collagen-induced arthritis (CIA) model mice. Changes in body weight **b**, paw score **c**, and paw thickness **d** after collagen immunization. The thickness of the hind paws was measured 10 weeks before sacrifice. The bars represent the mean ± SEM (*n* = 4, *p < 0.05, **p < 0.01, ***p < 0.001, ^#^*p* < 0.05, and ^###^*p* < 0.001; *: comparison between the CIA and CIA + S0859 groups, ^#^: comparison of the CIA + S0859 group between week 8 and week 10). S0859 was administered at the dose of 800 ng of S0859/g (weight). **e** Representative images of hind paws in the control, CIA, CIA + S0859, and S0859 groups at 6 (initial S0859 injection) and 10 (before sacrifice) weeks.

Next, to evaluate the therapeutic effect of S0859 in an animal model, we established a CIA mouse model and assessed structural abnormalities after 10 weeks of arthritis development. A flow chart of the developmental procedure for CIA is shown in Fig. 5a. Arthritis was induced in DBA/1 mice. Bodyweight was maintained after the booster injection in CIA mice, whereas body weight gradually increased in the control group (Fig. 5b). At week 8, CIA mice lost weight, whereas the weight of CIA mice treated with S0859 started to recover (Fig. 5b). The arthritis severity determined by the paw score during the developmental period (Fig. 5c) and the changes in hind paw thickness at 10 weeks (Fig. 5d) were greater in CIA mice than in control mice. The S0859-treated CIA group showed reduced paw scores and hind paw thickness (Fig. 5c, d). Pathologic swelling of the paws was also observed in CIA mice, whereas the S0859-treated CIA group showed fewer swollen paws at 10 weeks (Fig. 5e). We verified the results with comparisons to the vehicle (DMSO)-injected groups of control and CIA mice (Supplementary Fig. 10). These results suggest that treatment with S0859 attenuates the structural abnormalities observed in the paws and bones in the CIA group.

### S0859 reduced bone erosion and edema in the joints of CIA mice

**Fig. 6:**
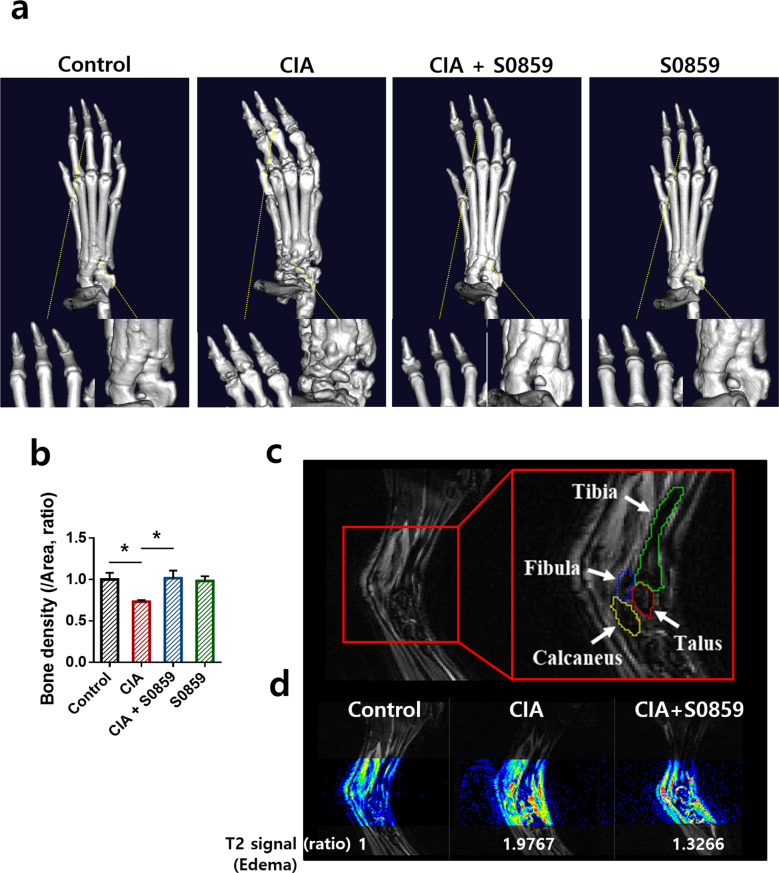
S0859 reduced bone erosion and edema in the joints of CIA mice. **a** Three-dimensional (D) micro-CT imaging of the hind paw in the CIA mouse model with or without 800 ng of S0859/g (weight) treatment. **b** Bone density determined from 2D micro-CT images. The bars represent the mean ± SEM (*n* = 6, *p < 0.05). **c** Morphological comparison of normal and CIA mice using magnetic resonance imaging (MRI). Segmentation of regions of interest, the tibia (green), the fibula (blue), the talus (red), and the calcaneus (yellow) in a normal mouse using a T2-weighted image. **d** T2 signal intensity of ankle edema in each group. The ratio of the T2 signal is presented.

Additionally, to evaluate the therapeutic effect of S0859 in CIA-induced mice, bone erosion was determined by micro-CT analysis. Enhanced bone erosion and rough bone surfaces in CIA mice were reduced by treatment with S0859 (Fig. 6a, b). We verified the results with comparisons to the vehicle-injected groups of control and CIA mice. Vehicle alone did not affect the structural changes observed at 6 and 8 weeks in control and CIA mice, respectively, and the severity of CIA did not change in the presence of vehicle (Supplementary Figs. 11 and 12). We also performed morphometric analysis of a region of interest using an MRI technique. T1-weighted and T2-weighted images using MRI were cross-compared to manually segment each region of interest (tibia, fibula, calcaneus, and talus) in the joint area (Fig. 6c). In addition, we measured the T2 signal to evaluate arthritic disease in the mouse ankle in the form of a color scale (Fig. 6d). A stronger T2 signal, expressed in rainbow pseudocolor, indicated severe edema, while a weaker T2 signal, expressed in blue (baseline), indicated less to no edema. The T2 signal on T2-weighted images for the control mouse group was weaker than that in the images for the other groups, with CIA mice showing a 1.98-fold higher T2 signal. Treatment with S0859 reduced the T2 signal of the joint area in CIA mice (Fig. 6d).

### S0859 reduced RA severity, NBC expression, and cytokine levels in CIA mice with no hepatic or renal toxicity

**Fig. 7:**
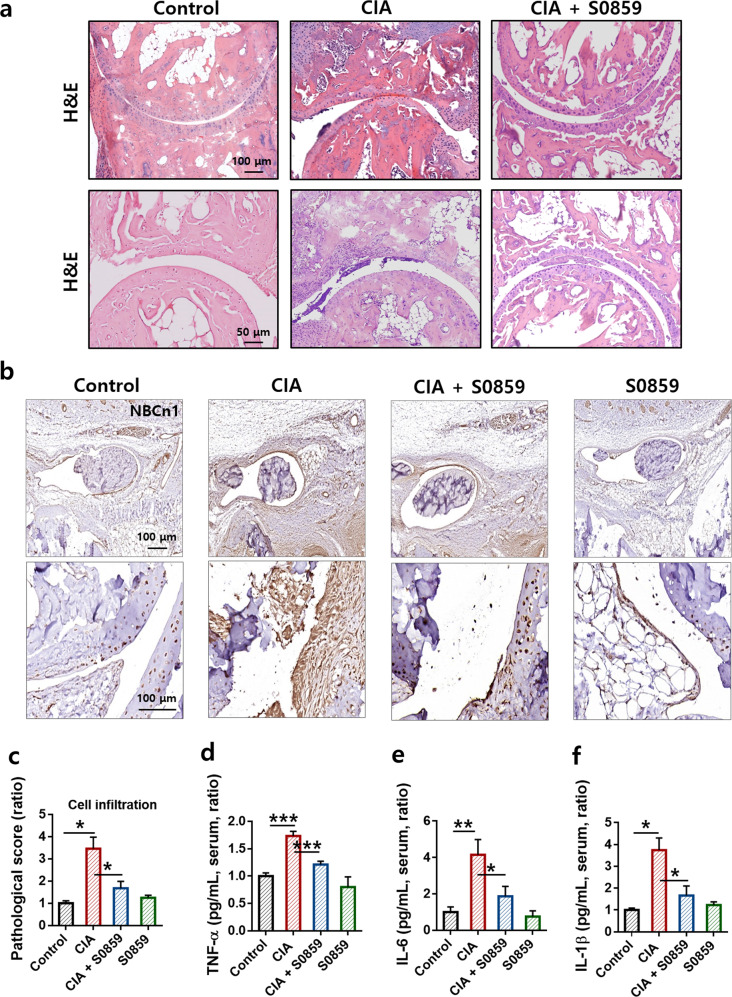
S0859 reduced RA severity, NBC expression, and cytokine levels in CIA mice with no hepatic or renal toxicity. **a** Histological changes in the joint and synovial tissues as assessed by hematoxylin and eosin (H&E) staining and **b** immunohistochemical staining with an anti-NBCn1 antibody. The top panels in **b** represent magnified images of tissue near the talus and navicular. The bottom panels in **b** represent images of the joint between the talus and calcaneus. The scale bars represent 100 μm or 50 μm. **c** Pathological score for cell infiltration determined by H&E staining. The bars represent the mean ± SEM (*n* = 3, *p < 0.05). Measurement of TNF-α **d**, IL-6 **e**, and IL-1β **f** levels in the serum of CIA mice using ELISA, and the levels are represented as ratios. The bars represent the mean ± SEM (*n* = 3, *p < 0.05, **p < 0.01, and ***p < 0.001).

Treatment with S0859 reduced NBCn1 expression and the number of infiltrated cells in the synovial tissues of CIA mice (Fig. 7a–c). NBCn1 expression was enhanced in the connective tissue near the talus and navicular (Fig. 7b, images of the joints between the talus and calcaneus are shown in the bottom panel). The levels of inflammatory cytokines, including TNF-α, IL-1β, and IL-6, were also reduced in CIA mice in the presence of S0859 (Fig. 7d–f). We further evaluated the results of a preclinical analysis with S0859. The CBC test checked the HGB, HCT, MCH, MCHC, MCV, and PLT levels (Supplementary Fig. 13). The blood composition, such as RBC, WBC, EOS, NEU, and LYM counts, did not change among the groups (Supplementary Fig. 14). The hepatic and renal toxicities of S0859 were negligible in all groups, as indicated by analyzing the levels of ALT, AST, T-Bili (Supplementary Fig. 15a–c), and BUN (Supplementary Fig. 15d).

### S0859 attenuated the migration of primary cultured FLSs from CIA mice

**Fig. 8:**
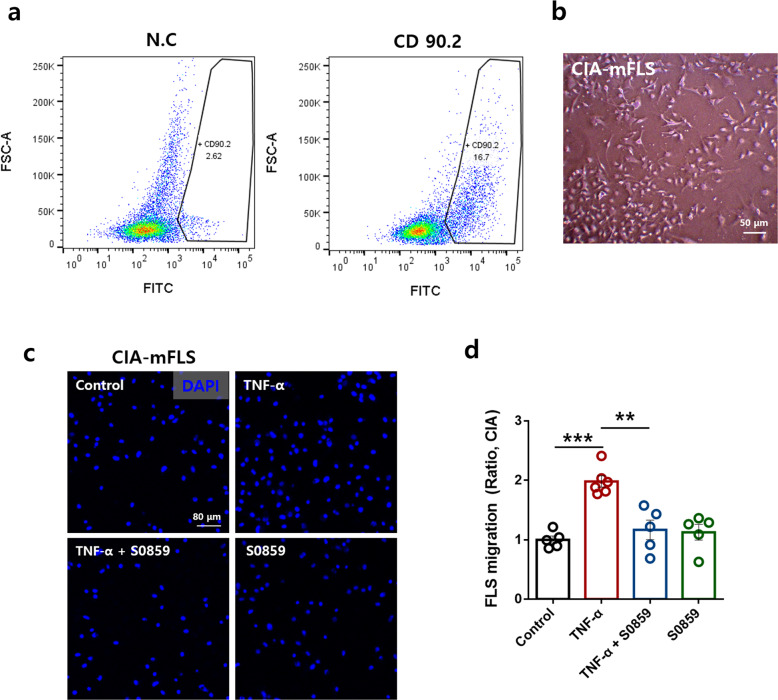
S0859 attenuated the migration of primary cultured FLSs from CIA mice. **a** FACS scatter plots showing the CD90.2-positive cell population, marked as FITC positive, in FLSs isolated from CIA mice. N.C: negative control. **b** Morphology of primary cultured FLSs isolated from CIA mice (CIA-mFLSs). The scale bar represents 50 μm. **c** Primary cultured CIA-mFLS migration was measured in the presence of 20 μM S0859 in the Transwell culture system. The scale bar represents 80 μm. **d** Analysis of the total DAPI intensity to assess FLS migration. The bars represent the mean ± SEM (*n* = 5–6, **p < 0.01 and ***p < 0.001).

To understand whether the migration of inflamed RA-FLSs can be reduced by NBCs, we isolated FLSs from CIA mice (CIA-mFLSs). The isolated FLSs were identified with CD90.2^33^, an FLS marker (Fig. 8a), and images were acquired (Fig. 8b). Treatment with S0859 attenuated the migration of primary cultured CIA-mFLSs (Fig. 8c, d).

## Discussion

To the best of our knowledge, this is the first study to show that NBCn1 is recruited in FLSs in response to inflamed synovial fluid and is involved in FLS migration. Moreover, treatment with the NBC inhibitor S0859 attenuated histological abnormalities and serum inflammatory cytokine levels in a CIA mouse model. We hypothesized that NBCn1 can be recruited in response to physical and biochemical stimuli, such as an acidic pH or inflammatory cytokines, respectively, in RA-FLSs.

Inflamed synovial fluid contains various inflammatory cytokines^8,40^. We hypothesized that inflammatory stimuli induce cellular mobility via the recruitment of transporters toward the stimuli. This unique concept for the modulation of ion transporters drives the application of a treatment strategy for RA. Inflamed synovial fluid and the inflammatory cytokine TNF-α are sufficient stimuli to activate FLS migration. Our study showed that the involvement of NBCn1 in the stimulation conveyed by inflamed synovial fluid was related to FLS migration and that inhibition of NBCn1 activity by an NBCn1 inhibitor reduced RA severity in a CIA mouse model.

FLSs are considered contributors involved in modulating the composition of synovial fluids as well as producers of aggressive inflammatory signals and mediators of joint destruction^45,46^. Stimulation with cytokines and chemokines induces cellular migration, including the migration of immune cells, such as monocytes, and FLSs, although the migratory mechanism is not limited to specific cells. Recently, the involvement of gastrin-releasing peptide (GRP)^47^, adrenomedullin^48^, follistatin-like protein 1 (FLP 1)^44^, cadherin 11^49,50^, and huntingtin-interacting protein 1 in FLS invasion-related signaling has been addressed^51^. Therapeutic strategies have been employed to attenuate FLS invasion through the application of molecules and drugs, such as GRP receptor antagonists^47^ and microRNA targets of FLP 1^15^.

**Fig. 9:**
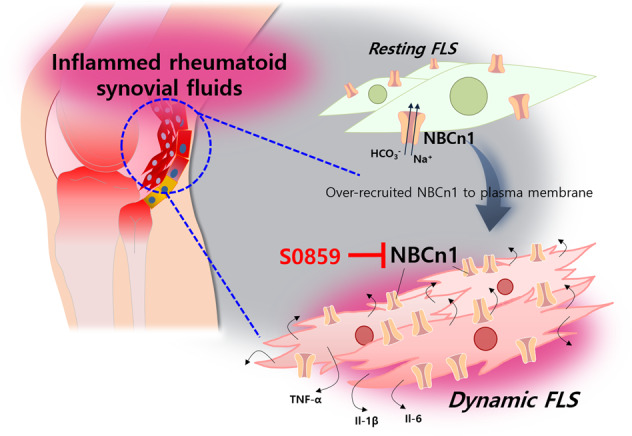
A schematic model of RA in the presence of S0859. NBCn1 recruited to the plasma membrane of resting FLSs converted the cells into dynamic FLSs through the inflammatory signals in the synovial fluid of inflamed joints. This mechanism was attenuated by the NBC inhibitor S0859.

The migratory mechanism is physically derived from the recruitment of ion transporters^12^. Most studies on migratory mechanisms have focused on cancer metastasis and wound healing^52–54^. Ion transporters have been reported to drive the directional movement of modules toward stimuli during wound healing^55^. Thus, we speculated that various inflammatory input-mediated cellular migration processes exhibit enhanced directionality through the involvement of migratory modules. Although the stimuli, such as cytokines and chemokines, are varied, the strategy of developing an attenuated migratory module seems to be relevant for modulating RA. Modulation of the migratory module would be an effective strategy for inhibiting migration to expand additional inflammatory signaling. Stimulation with inflammatory cytokines, such as TNF-α and IL-17, mechanically induces cellular movement through the recruitment of the NBC ion transporters, especially NBCn1, to the plasma membrane of FLSs. We provided a schematic model of RA showing the involvement of dynamic FLSs, which exhibit NBCn1 recruitment in the inflamed synovium (Fig. 9). Additionally, NBCn1 is the main contributor to cellular movement in breast cancer metastasis and renal epithelial cell migration^19,22,56^. We also previously reported that the membrane recruitment of NBCn1 provides the migratory machinery of cancer cells^34^. Among the NBC family members, NBCn1 is a dominant contributor to TNF-α signaling in FLSs, as shown in Fig. 1c. The involvement of NBCn1 contributes to the similar features between cancerous and inflamed synovial tissues, such as those observed under acidic and hypoxic conditions^57^.

Overall, NBCn1, which is present in inflamed synovial tissues, especially in FLS, is involved in cellular migration. Therapeutic targeting of NBCn1 could be an innovative strategy for attenuating RA severity. The enhanced NBCn1 activity in RA-FLSs reflects the recruitment of the migratory machinery induced by stimulation with inflamed synovial fluid or inflammatory mediators, such as TNF-α and IL-17. Our results will provide insight for the development of a more effective strategy for RA treatment to enhance the small incremental benefits of current clinical strategies, including various anti-cytokine therapies (anti-TNF agents and IL inhibitors), immune suppressants, and disease-modifying anti-rheumatic drugs^58,59^. Inhibition of NBCn1 by S0859 attenuated FLS migration, exerted an inhibitory effect on joint destruction and reduced inflammatory cytokine levels in mouse serum without exhibiting hepatic or renal toxicity, suggesting that the identification of NBCn1 as a newly developed therapeutic target may lead to a migratory module-based strategy for RA therapy.

## Supplementary information

Supplementary materials

## Animal studies

All experimental animal procedures were performed in accordance with the Gachon University guidelines and were approved by the Animal Care and Use Committee of Gachon University (LCDI-2019-0139).
