# Supplementary material for: Dynamic synovial fibroblasts are modulated by NBCn1 as a potential target in rheumatoid arthritis
Source: Exp Mol Med. 2022 Apr 12;54(4):503–17. doi: 10.1038/s12276-022-00756-6 (PMC9076869; doi:10.1038/s12276-022-00756-6)
Supplement: Supplementary file 1 — Supplementary materials [file 12276_2022_756_MOESM1_ESM.pdf]

Dynamic synovial fibroblasts are modulated by NBCn1 as a potential target in rheumatoid arthritis

Supplementary Table 1. Ji et al.

Genes	Sequences (5′→3′)
Human SLC4A4	(Forward) ACA ATG ATG ATG AGA AAG ATC ACC A<br>(Reverse) ACT TGG CAT ACC GAG TGA CTG
Human SLC4A7	(Forward) AAT TCC TAC GGG TGC TGA GG<br>(Reverse) GTA AGG AGG ACA GCA GGA GC
Human SLC4A8	(Forward) GAG TGA TCT GCT CAG ACC CG<br>(Reverse) CTC TGA TAG CTG AGG ACG CC
Human SLC4A10	(Forward) GGT GCT TAT TCC AGA GGC GT<br>(Reverse) ATT ATC CGT AGG CAG CAG CG
Human IL-6	(Forward) CCC CCA GGA GAA GAT TCC AA<br>(Reverse) CCG TCG AGG ATG TAC CGA ATT
Human IL-1β	(Forward) CCA CGG CCA CAT TTG GTT<br>(Reverse) AGG GAA GCG GTT GCT CAT C
Human TNF-α	(Forward) CAA GCC TGT AGC CCA TGT TGT<br>(Reverse) TTG GCC AGG AGG GCA TT
Human MMP-3	(Forward) CCC TCC AAC CGT GAG GAA AA<br>(Reverse) GCT AAG CAG CAG CCC ATT TG
Human MMP-9	(Forward) CGC TGG GCT TAG ATC ATT CC<br>(Reverse) GTG CCG GAT GCC ATT CA
Human MMP-13	(Forward) GAG TTC GGC CAC TCC TTA GGT<br>(Reverse) GCT TTT GCC GGT GTA GGT GTA
Human MMP-14	(Forward) CCG GCC TTC TGT TCC TGA TA<br>(Reverse) GGT GTC AAA GTT CCC GTC ACA
Human DKK-1	(Forward) TGG AAC TCC CCT GTG ATT GC<br>(Reverse) TGG AAC TCC CCT GTG ATT GC
Human GAPDH	(Forward) GAC CTG ACC TGC CGT CTA GAA A<br>(Reverse) CCT GCT TCA CCA CCT TCT TGA

Supplementary Table 1. Primers list

Footnotes: SLC4A4/7/8/10: solute carrier family 4 member 4/7/8/10; IL-6/1β: interleukin-6/1β; TNF-α: Tumor necrosis factor-α; MMP-3/9/13/14: matrix metalloproteinase-3/9/13/14; DKK-1: dickkopf-1; GAPDH: glyceraldehyde 3-phosphate dehydrogenase.

Supplementary Fig. 1. Ji et al.

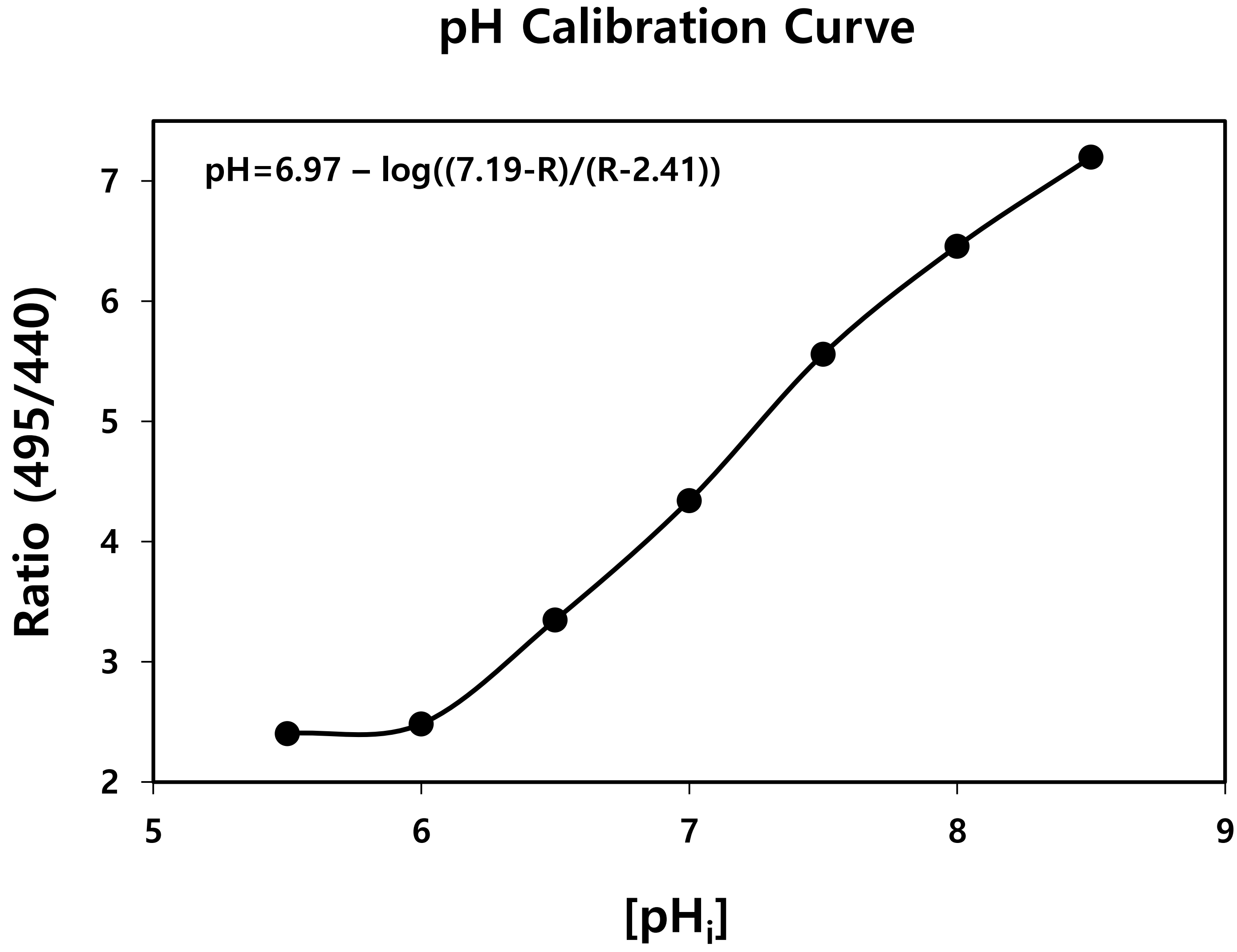

Supplementary Fig. 1. pH calibration curve for FLS at pH 5.5, 6.0, 6.5, 7.0, 7.5, 8.0, and 8.5. The equation of the calibrated curve is presented.

Supplementary Fig. 2. Ji et al.

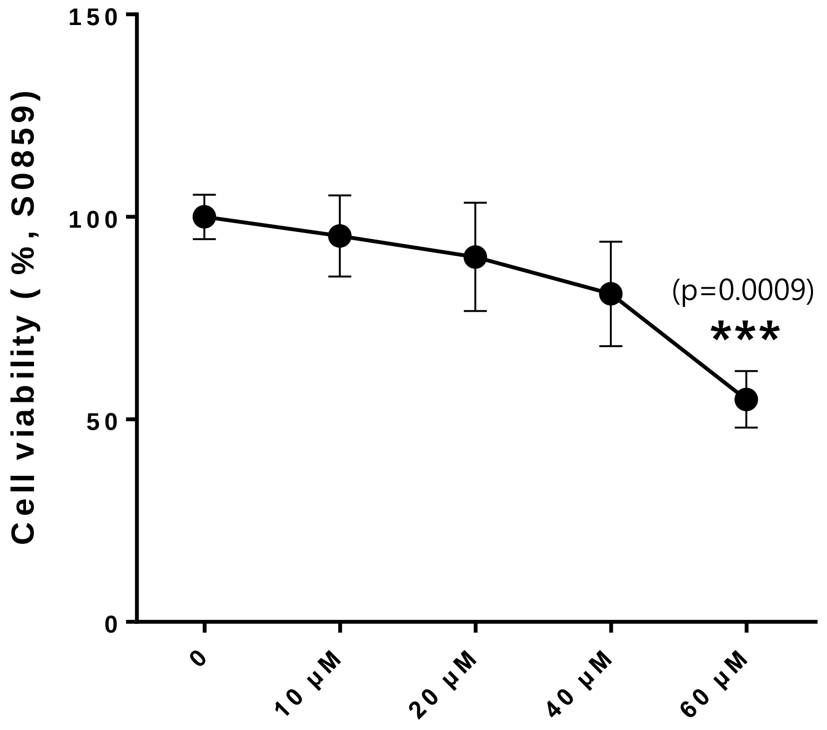

Supplementary Fig. 2. Cell viability was determined by MTT assay with different doses of S0859 (0, 10, 20, 40, and 60 μM) in RA-FLS cells. Bars represent the mean ± SEM (n = 3, ***p < 0.001).

Supplementary Fig. 3. Ji et al.

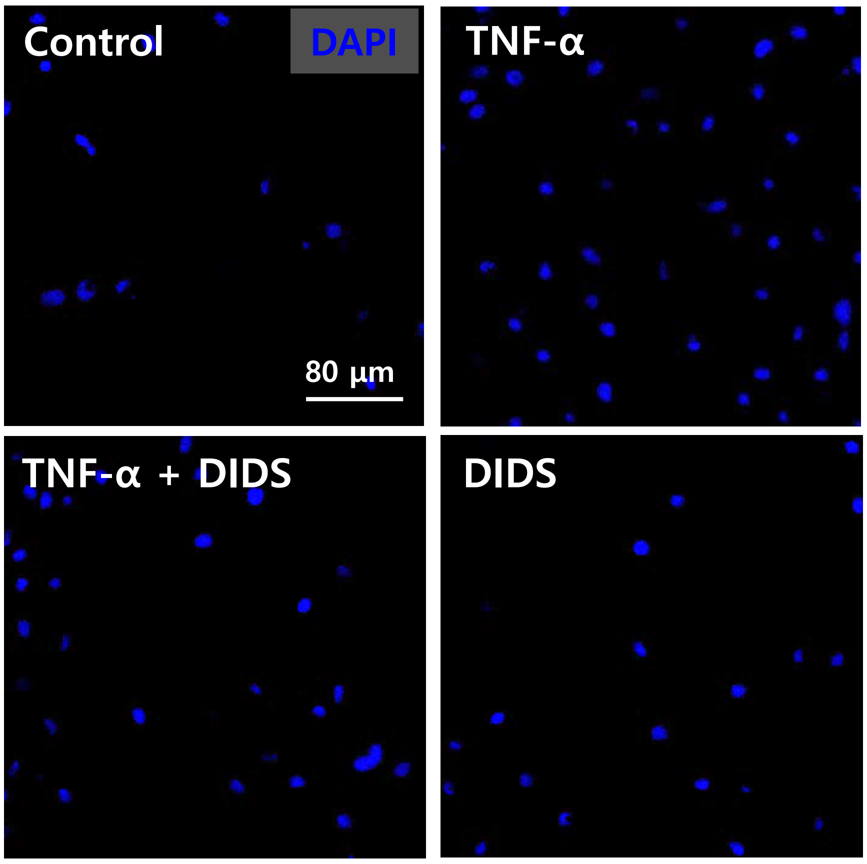

Supplementary Fig. 3. Immunofluorescence staining of DAPI (blue) after adding 10 ng/mL TNF-α to the bottom plate, and 500 μM DIDS to the upper chamber for 6 hrs. The scale bar represents 80 μm.

Supplementary Fig. 4. Ji et al.

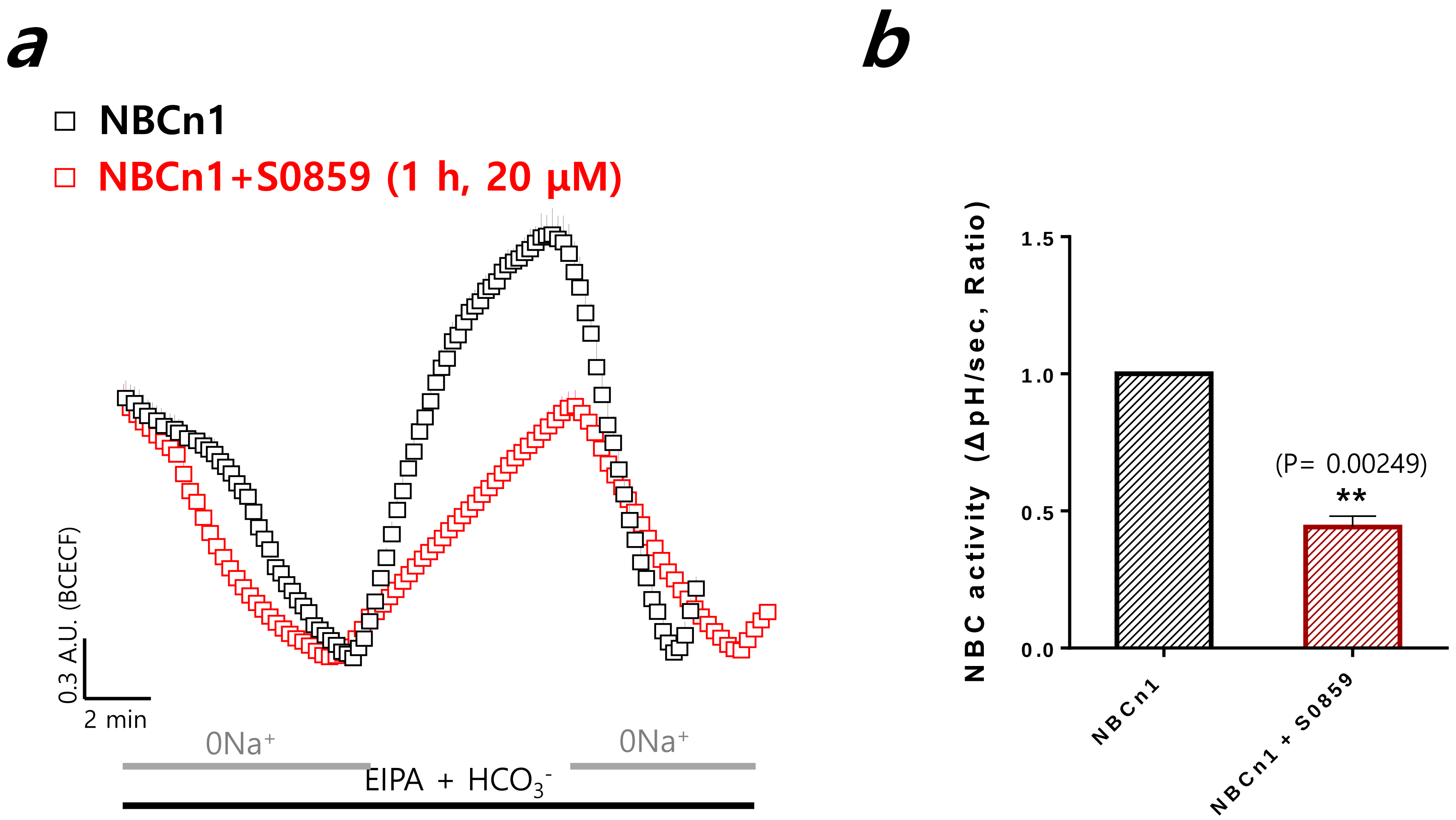

Supplementary Fig. 4. (a) NBC activity was assessed by measuring changes in pH_i in HEK293T cells transfected with NBCn1 plasmid with or without 20 μM S0859. **(b)** Bars represent the mean ± SEM (n = 3, **p < 0.01).

Supplementary Fig. 5. Ji et al.

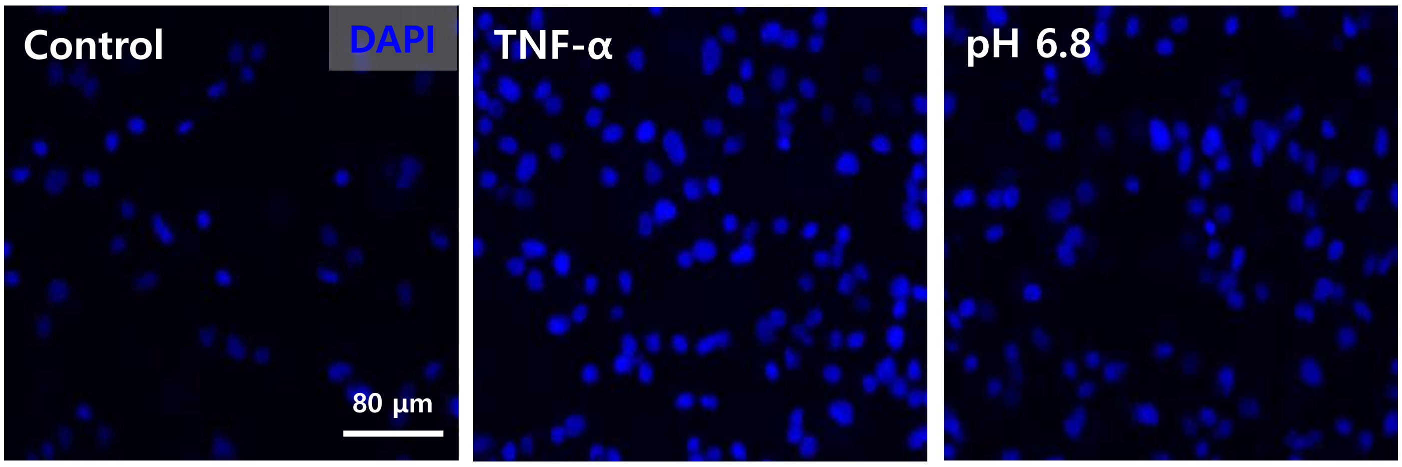

Supplementary Fig. 5. FLS migration was measured by DAPI staining (blue) with 10 ng/mL TNF-α or pH 6.8-conditioned media. The scale bar represents 80 µm.

Supplementary Fig. 6. Ji et al.

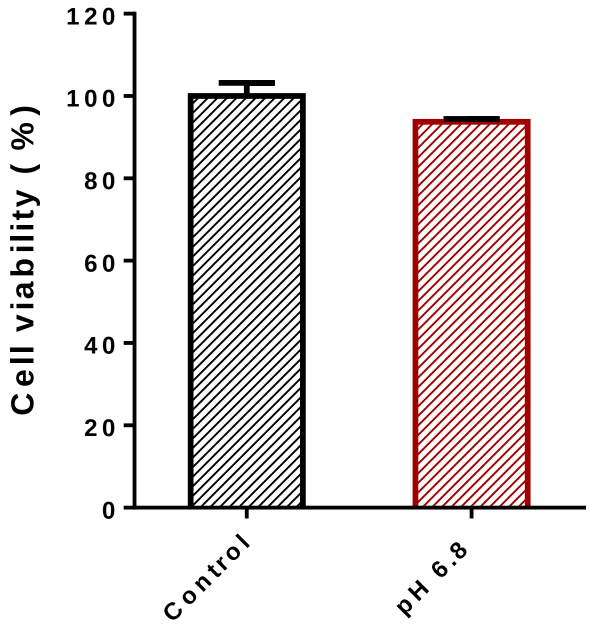

Supplementary Fig. 6. Cell viability of FLS was determined by MTT assay with pH 6.8-conditioned media. Bars represent the mean ± SEM (n = 3).

Supplementary Fig. 7. Ji et al.

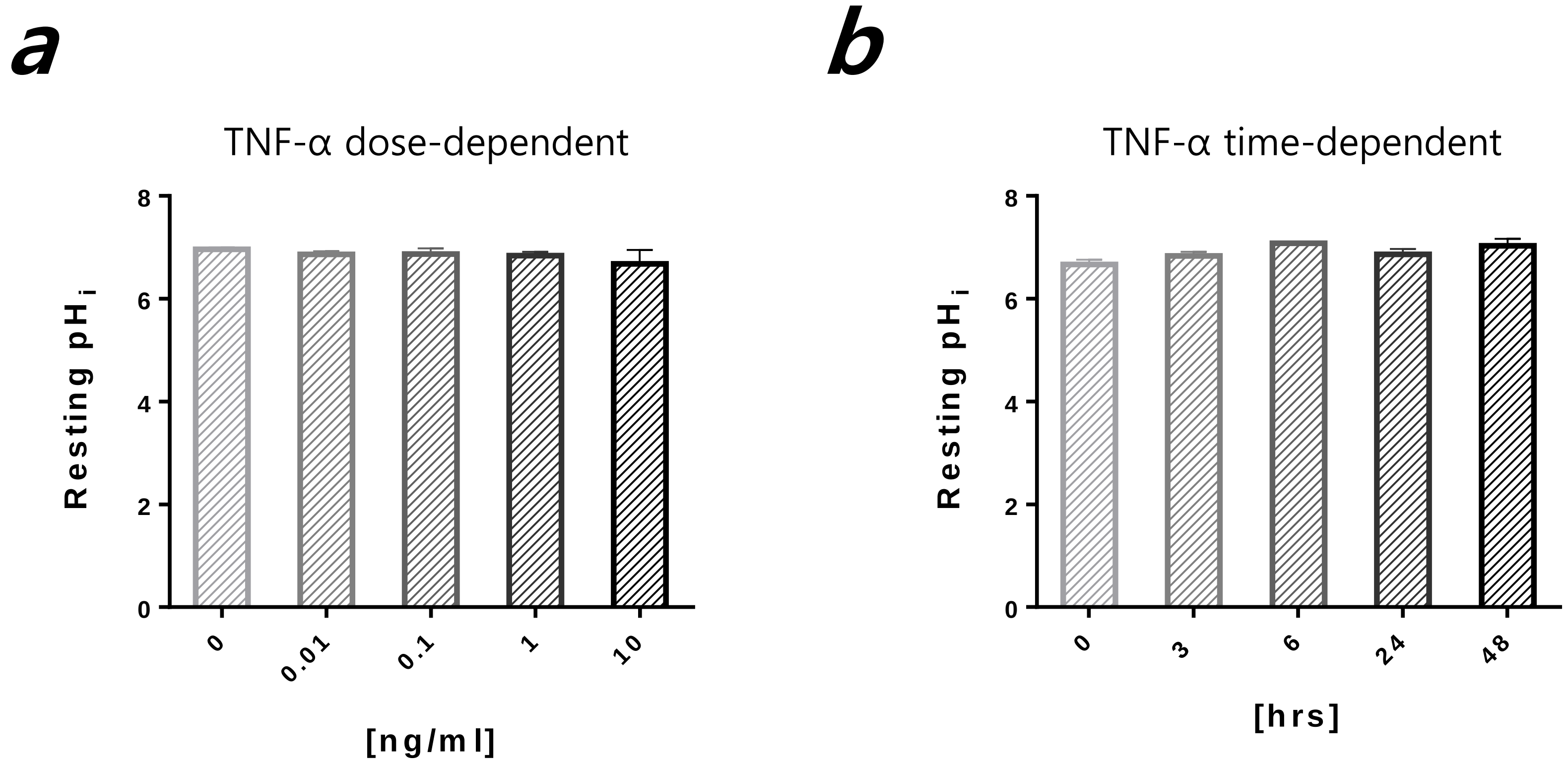

Supplementary Fig. 7. Resting pH_i levels of RA-FLS treated with TNF-α in **(a)** a dose- (for 24 hrs, 0, 0.01, 0.1, 1, and 10 ng/mL) and **(b)** time-dependent (at 10 ng/mL) manner.

Supplementary Fig. 8. Ji et al.

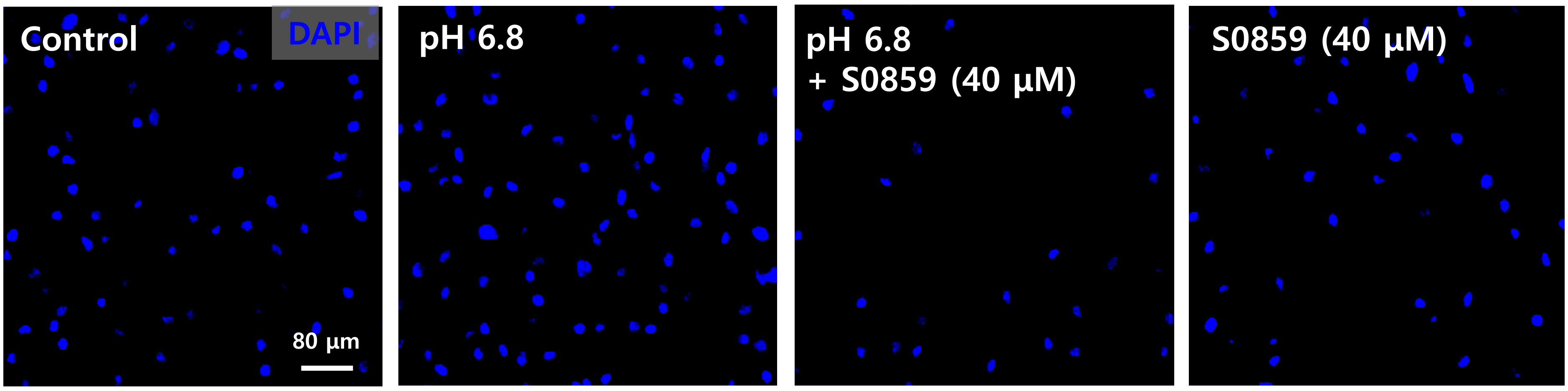

Supplementary Fig. 8. Immunofluorescence staining of DAPI (blue) in pH 6.8-conditioned media to the bottom plate, and 40 μM S0859 to the upper chamber for 6 hrs. The scale bar represents 80 μm.

Supplementary Fig. 9. Ji et al.

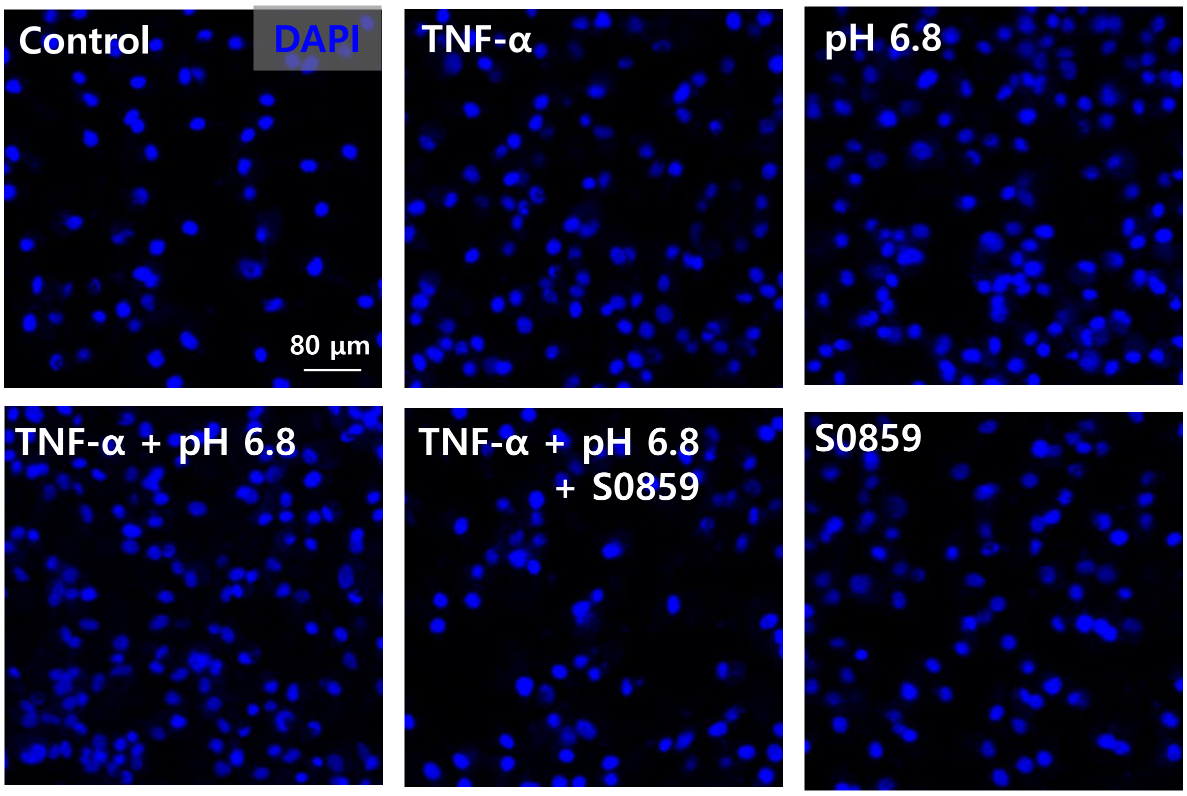

Supplementary Fig. 9. Immunofluorescence staining of DAPI (blue) in 10 ng/mL TNF-α, pH 6.8-conditioned media, or TNF-α+pH 6.8-conditioned media to the bottom plate, and 20 μM S0859 to the upper chamber for 6 hrs. The scale bar represents 80 μm.

Supplementary Fig. 10. Ji et al.

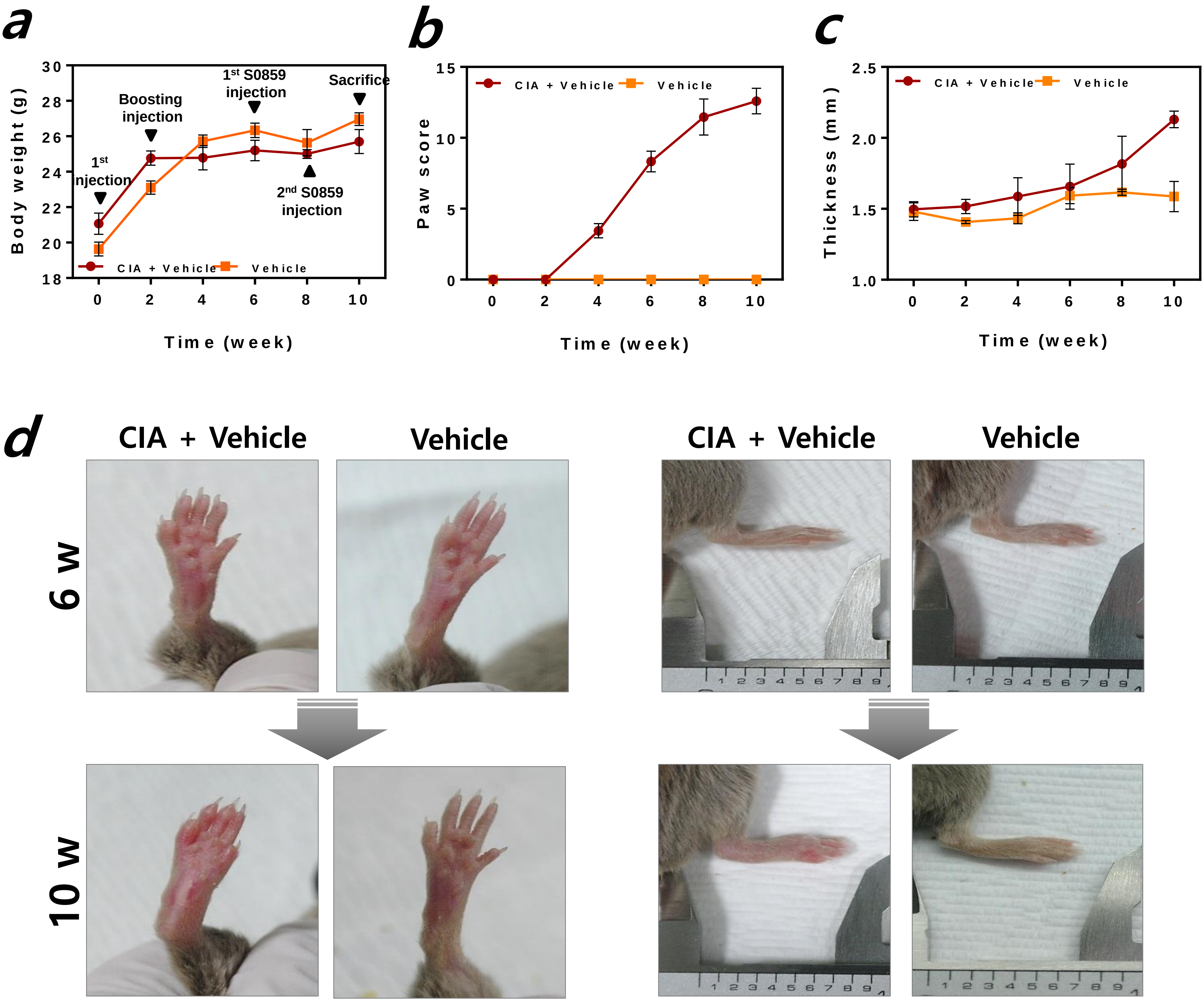

Supplementary Fig. 10. Changes in body weight **(a)**, paw score **(b)**, and hind paw thickness **(c)** after collagen immunization. The thickness of the hind paws was measured 10 weeks before sacrifice. **(d)** Representative images of the hind paws in CIA with vehicle and vehicle only groups at 6 (Initial drug injection) or 10 (before sacrifice) weeks.

Supplementary Fig. 11. Ji et al.

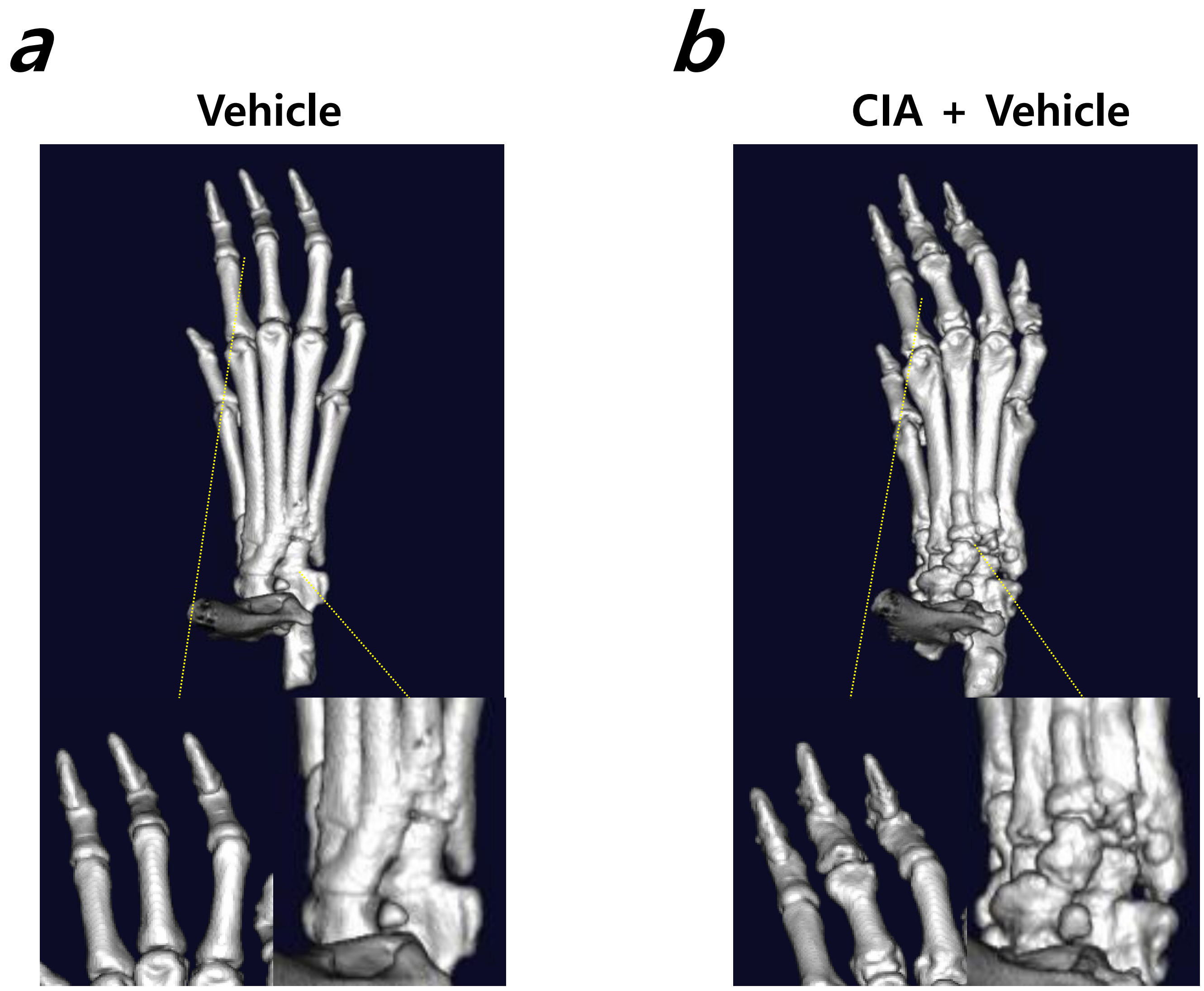

Supplementary Fig. 11. Three-dimensional micro-CT imaging of the hind paw in CIA mouse model with **(a)** vehicle (DMSO) and **(b)** CIA + vehicle at 10 weeks.

Supplementary Fig. 12. Ji et al.

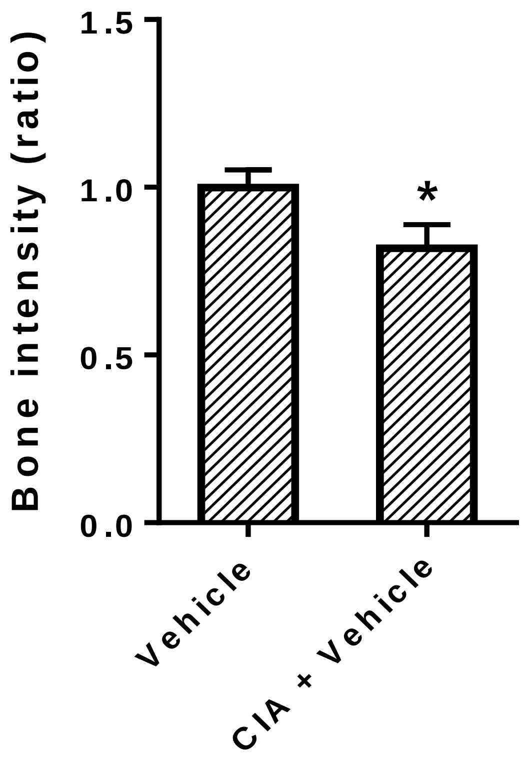

Supplementary Fig. 12. Bone density of control and CIA mice with vehicle from 2D-micro-CT images. The bars represent the mean ± SEM (n = 6, *p < 0.05).

Supplementary Fig. 13. Ji et al.

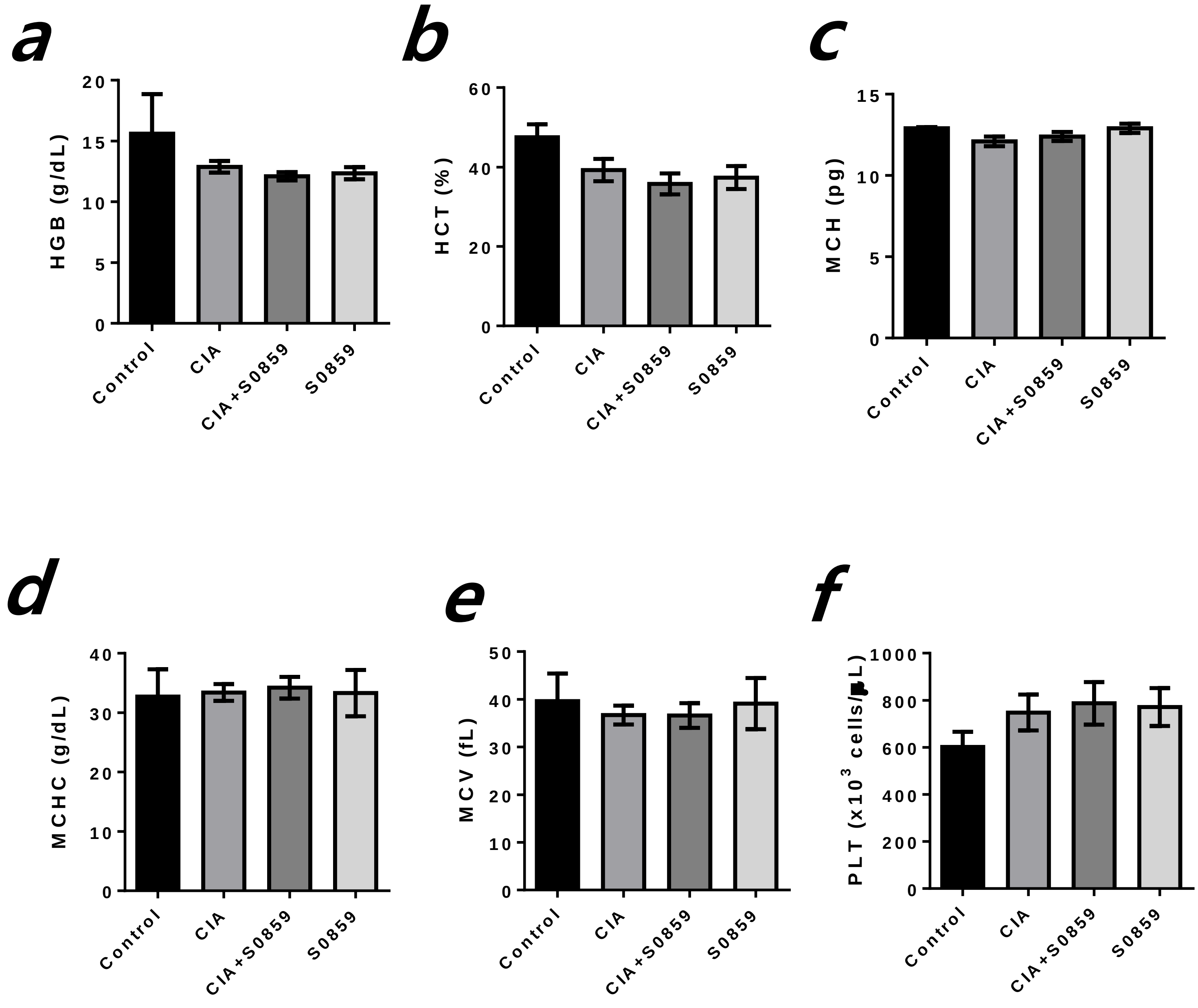

Supplementary Fig. 13. Comparison of complete blood counts in 8 mg/kg CIA mice with or without 0.8 mg/kg S0859. **(a)** Hemoglobin (HGB, g/dL), **(b)** Hematocrit (HCT, %), **(c)** Mean corpuscular hemoglobin (MCH, pg), **(d)** Mean corpuscular hemoglobin concentration (MCHC, g/dL), **(e)** Mean corpuscular volume (MCV, fL), and **(f)** Platelets (PLT, cells/μL).

Supplementary Fig. 14. Ji et al.

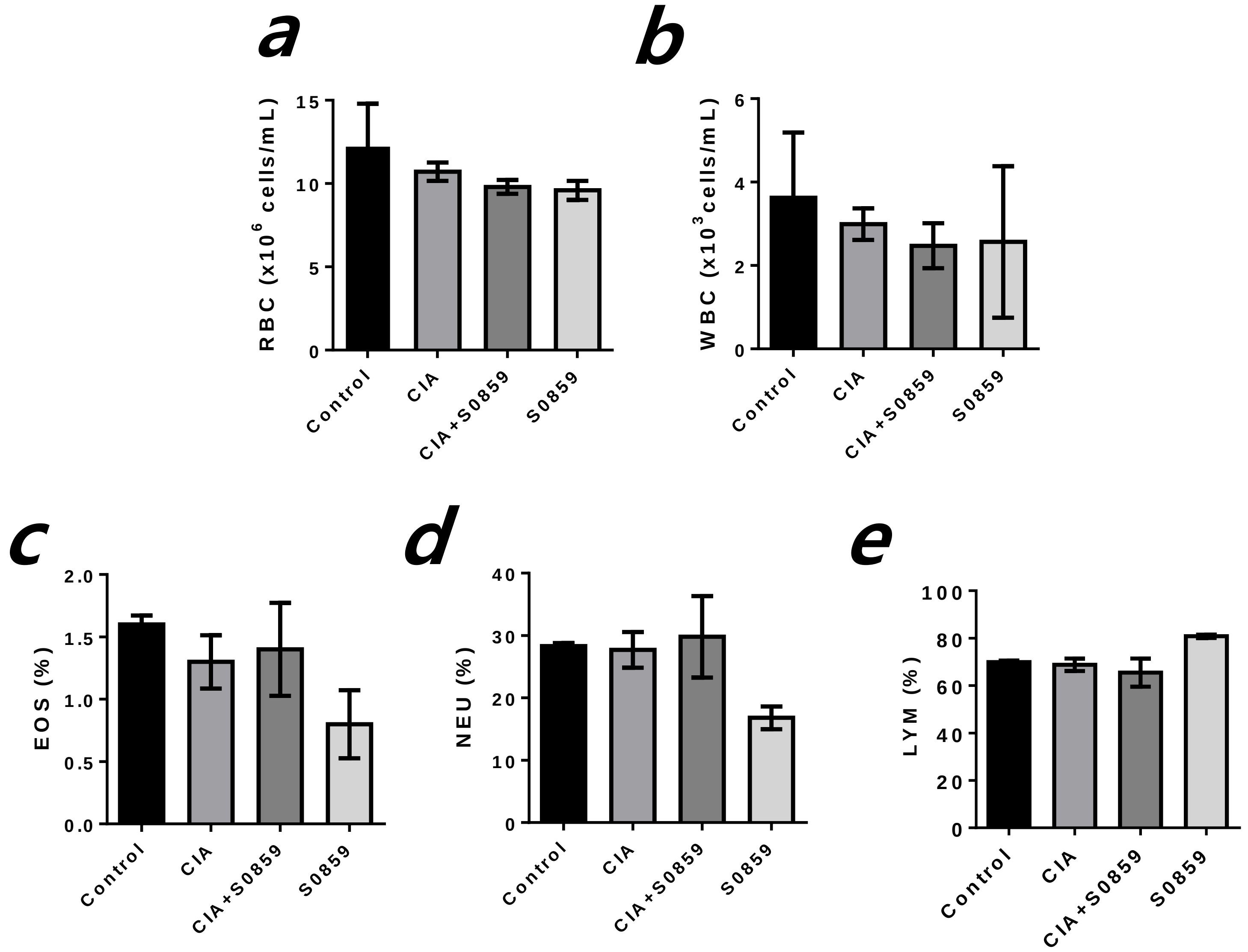

Supplementary Fig. 14. Comparison of complete blood counts (CBC) in 8 mg/kg CIA mice with or without 0.8 mg/kg S0859. **(a)** Red blood cells (RBC, cells/mL), **(b)** White blood cells (WBC, cells/mL), **(c)** Eosinophils (EOS, %), **(d)** Neutrophils (NEU, %), and **(e)** Lymphocytes (LYM, %).

Supplementary Fig. 15. Ji et al.

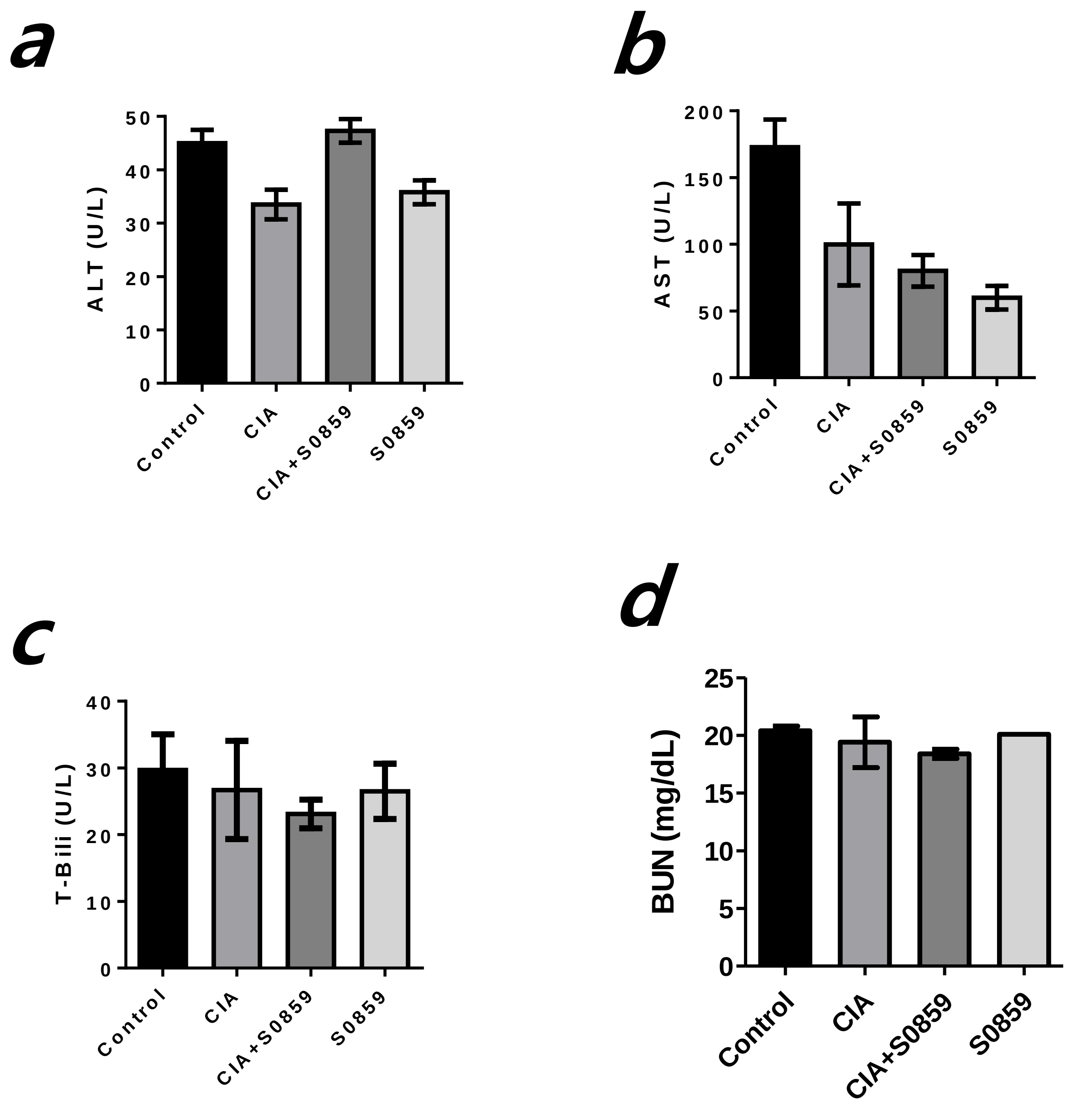

Supplementary Fig. 15. Comparison of hepatic and renal toxicity tests in 8 mg/kg CIA mice with or without 0.8 mg/kg S0859. Levels of **(a)** alanine aminotransferase (ALT, U/L), **(b)** aspartate aminotransferase (AST, U/L), **(c)** total bilirubin (T-Bili, U/L), and **(d)** blood urea nitrogen (BUN, mg/dL).
